# Natural Polymorphisms in *Tap2* Influence Negative Selection and CD4∶CD8 Lineage Commitment in the Rat

**DOI:** 10.1371/journal.pgen.1004151

**Published:** 2014-02-20

**Authors:** Jonatan Tuncel, Sabrina Haag, Anthony C. Y. Yau, Ulrika Norin, Amelie Baud, Erik Lönnblom, Klio Maratou, A. Jimmy Ytterberg, Diana Ekman, Soley Thordardottir, Martina Johannesson, Alan Gillett, EURATRANS Consortium, Pernilla Stridh, Maja Jagodic, Tomas Olsson, Alberto Fernández-Teruel, Roman A. Zubarev, Richard Mott, Timothy J. Aitman, Jonathan Flint, Rikard Holmdahl

**Affiliations:** 1 Section for Medical Inflammation Research, Department of Medical Biochemistry and Biophysics, Karolinska Institutet, Stockholm, Sweden; 2 Wellcome Trust Centre for Human Genetics, Oxford, United Kingdom; 3 Physiological Genomics and Medicine Group, Medical Research Council Clinical Sciences Centre, Faculty of Medicine, Imperial College London, Hammersmith Hospital, London, United Kingdom; 4 Rheumatology Unit, Department of Medicine, Karolinska University Hospital, Stockholm, Sweden; 5 Medical Proteomics, Department of Department of Medical Biochemistry and Biophysics, Karolinska Institutet, Stockholm, Sweden and Science for Life Laboratory, Solna, Sweden; 6 Department of Clinical Neuroscience, Karolinska Institutet, Neuroimmunology Unit, Center for Molecular Medicine, Karolinska University Hospital, Stockholm, Sweden; 7 Medical Psychology Unit, Department of Psychiatry & Forensic Medicine, Institute of Neurosciences, School of Medicine, Autonomous University of Barcelona, Bellaterra, Barcelona, Spain; Georgia Institute of Technology, United States of America

## Abstract

Genetic variation in the major histocompatibility complex (MHC) affects CD4∶CD8 lineage commitment and MHC expression. However, the contribution of specific genes in this gene-dense region has not yet been resolved. Nor has it been established whether the same genes regulate MHC expression and T cell selection. Here, we assessed the impact of natural genetic variation on MHC expression and CD4∶CD8 lineage commitment using two genetic models in the rat. First, we mapped Quantitative Trait Loci (QTLs) associated with variation in MHC class I and II protein expression and the CD4∶CD8 T cell ratio in outbred Heterogeneous Stock rats. We identified 10 QTLs across the genome and found that QTLs for the individual traits colocalized within a region spanning the MHC. To identify the genes underlying these overlapping QTLs, we generated a large panel of MHC-recombinant congenic strains, and refined the QTLs to two adjacent intervals of ∼0.25 Mb in the MHC-I and II regions, respectively. An interaction between these intervals affected MHC class I expression as well as negative selection and lineage commitment of CD8 single-positive (SP) thymocytes. We mapped this effect to the transporter associated with antigen processing 2 (*Tap2*) in the MHC-II region and the classical MHC class I gene(s) (*RT1-A*) in the MHC-I region. This interaction was revealed by a recombination between *RT1-A* and *Tap2*, which occurred in 0.2% of the rats. Variants of *Tap2* have previously been shown to influence the antigenicity of MHC class I molecules by altering the MHC class I ligandome. Our results show that a restricted peptide repertoire on MHC class I molecules leads to reduced negative selection of CD8SP cells. To our knowledge, this is the first study showing how a recombination between natural alleles of genes in the MHC influences lineage commitment of T cells.

## Author Summary

Peptides from degraded cytoplasmic proteins are transported via TAP into the endoplasmic reticulum for loading onto MHC class I molecules. TAP is encoded by *Tap1* and *Tap2*, which in rodents are located close to the MHC class I genes. In the rat, genetic variation in *Tap2* gives rise to two different transporters: a promiscuous A variant (TAP-A) and a more restrictive B variant (TAP-B). It has been proposed that the class I molecule in the DA rat (RT1-A^a^) has co-evolved with TAP-A and it has been shown that RT1-A^a^ antigenicity is changed when co-expressed with TAP-B. To study the contribution of different allelic combinations of *RT1-A* and *Tap2* to the variation in MHC expression and T cell selection, we generated DA rats with either congenic or background alleles in the *RT1-A* and *Tap2* loci. We found increased numbers of mature CD8SP cells in the thymus of rats which co-expressed RT1-A^a^ and TAP-B. This increase of CD8 cells could be explained by reduced negative selection, but did not correlate with RT1-A^a^ expression levels on thymic antigen presenting cells. Thus, our results identify a crucial role of the TAP and the quality of the MHC class I repertoire in regulating T cell selection.

## Introduction

Major histocompatibility complex (MHC) genes have been identified in all vertebrate species [1]. The 3.6 Mb human leukocyte antigen (HLA) was one of the first MHC to be sequenced, and revealed a region with extraordinary complexity [2]. The region contains ∼260 genes that are clustered in sub-regions denoted MHC-I, MHC-II and MHC-III [3]. Genes in the MHC were early recognized for their extreme sequence diversity and association with autoimmune and inflammatory conditions (reviewed in [4]). However, these associations have been difficult to delineate since nearly 40% of the MHC genes have immune-related functions [2]. The interpretation of association data is further complicated by the extensive linkage disequilibrium (LD) across the region [5]. While the LD structure [6], [7] and genetic variation [3], [8] of the HLA in humans is rather well investigated, similar detailed analysis for the MHC in other species is needed.

The first complete sequence of the rat MHC (RT1) on chromosome 20 was derived from the Brown Norway (BN) strain (RT1^n^) and released in 2004 [9]. The BN genome sequence, which is also the rat reference sequence (RefSeq), was published shortly thereafter [10]. Several inbred rat strains have since then been resequenced, including the Spontaneously Hypertensive Rat (SHR, RT1^k^) [11], and more recently, the DA (RT1^av1^), F344 (RT1^lv1^) [12] and a panel of additional strains [13]. The genetic organization of the MHC is similar in rats and humans, with the exception of a proximal classical MHC class Ia region (*RT1-A*) [14] and a larger number of telomeric, non-classical, class Ib genes (*RT1-CEM*) [15] in the rat. MHC class Ib molecules are structurally similar to class Ia molecules but the corresponding class Ib genes are less polymorphic, expressed at lower levels and several are pseudogenes [16], [17]. The number of functional class Ia genes (*RT1-A1, RT1-A2, RT1-A3*) in the rat varies between one and three in standard inbred strains [18], [19]. These genes are highly polymorphic and densely expressed on the surface of virtually all nucleated cells where they engage in presentation of intracellular antigens to cytotoxic CD8 T cells. The class Ia genes are surrounded by highly conserved framework genes, which are found in the MHC of all mammals, as well as several antigen processing and transportation genes [9]. To this latter group of genes belong the γ-interferon-inducible proteasome subunit beta type 8 (*Psmb8*) and *Psmb9*, and *Tap1* and *Tap2*, which are all located in the MHC-II region. An additional gene in this group, Tap binding protein (*Tapbp*), is located centromeric to the class Ia genes in the MHC-I region. These genes show limited allelic variation in humans and mice [20], [21]. In the rat, by contrast, allelic variants of *Tap2* fall into two groups, *Tap2A* and *Tap2B*, which encode two functionally distinct allotypic forms of Tap2 [22], [23]. Consequently, the two variants of TAP transporters encoded by *Tap1* and *Tap2A* or *Tap2B* are denoted TAP-A and TAP-B, respectively [19]. Analyses of inbred rat strains have revealed a significant degree of co-evolution between alleles in *Tap2* and the class Ia loci [19] and based on these studies RT1-A molecules have been classified as either TAP-A or TAP-B linked [19], [24]. Livingstone and colleagues have shown that if linkage is lost between *RT1-A^a^* (the DA allele of class Ia) and the *Tap2A* allele, as in the event of a recombination, the antigenicity of the class Ia molecule is altered [25]. This phenomenon, known as *class-I modification* (cim), has been explained by the peptide selectivity of the different TAP isoforms [26], [27] and more specifically by the inability of the TAP-B transporter to translocate peptides with C-terminal arginine residues, which are required for optimal loading of RT1-A^a^ molecules [28]. This was followed by studies showing that cim leads to abnormally slow assembly and reduced extracellular class I expression of RT1-A^a^ molecules [22], [29]. By contrast, no such effects have been described for TAP-B-linked RT1-A molecules when associated with the promiscuous TAP-A transporter. Within the MHC-II region are four additional antigen processing genes encoding the α and β subunits of RT1-DM and RT1-DO. These non-classical class II molecules are responsible for editing the peptide cargo of the classical class II molecules before these are translocated to the cell surface to present peptides to helper CD4 T cells.

Expression of MHC class I and II molecules on antigen presenting cells is required for T cell selection in the thymus [30], [31] and for the survival of mature T cell subsets in the periphery [32], [33]. The commitment of thymocytes to CD4 or CD8 lineage is determined mainly by the MHC peptide repertoire and the antigen specificity of the T cell receptor (TCR). The variation in CD4 and CD8 T cell numbers is associated with genes in the MHC in humans [34], mice [35] and rats [36]. However, it is not known whether the levels of MHC expression and/or polymorphisms in other genes than the classical MHC class I and II genes, such as the aforementioned antigen processing genes, contribute to this variation.

Here, we assessed the impact of the genetic variation on class I and II expression and T cell selection using two genetic rat models: the NIH-Heterogenous Stock (HS) and a panel of MHC-recombinant congenic strains. The NIH-HS is descended from eight inbred strains through 60 generations of outbreeding [37]. The greater genetic diversity in the HS compared to a conventional F2 cross allows mapping of more QTLs [38], which was exploited here to identify genomic regions (QTLs) that contribute both to MHC expression and the variation in CD4∶CD8 T cell ratio. Recombinant congenic strains (RCS) are powerful tools to resolve complex QTLs in regions with extensive LD. In RCS, haplotype blocks that are the result of rare recombinations can be preserved, which allows investigation of multiple phenotypes using genetically identical individuals [39], [40]. We describe a new panel of rat RCS, in which MHC segments from four inbred strains have been inserted on the background of the DA strain.

In the rat HS we identified a region spanning 4.1–9.7 Mb on chromosome 20 that contributed to both the variation in MHC expression and CD4∶CD8 T cell ratio. Mapping using RCS identified two intervals, each ∼0.25 Mb wide, in the MHC-I and II region that contributed to the phenotypic variation, suggesting that the HS QTL might result from two linked QTLs. First, we showed that the variation in class I expression correlated with alleles of *Tap*2, which is in line with previously reported data [25]. Second, we identified a novel type of class-I modification that we termed *inverse* cim, which reduced the expression of TAP-B linked RT1-A molecules if associated with TAP-A. Finally, we showed that polymorphisms in *Tap2* significantly contribute to CD8 lineage commitment, likely by altering the class-I peptide repertoire on antigen presenting cells in the thymus.

## Results

### QTLs for CD4∶CD8 T cell ratio and MHC expression overlap within the MHC region

T cell selection and the maintenance of the peripheral T cell pool rely on the interaction between peptide-MHC complexes and T cell receptors. The variation in the relative proportion of peripheral CD4 and CD8 T cells has been associated with the MHC [34]–[36]. In order to investigate if there is a shared genetic regulation of MHC expression and T cell numbers, we first identified genome-wide QTLs that controlled the CD4∶CD8 T cell ratio and MHC class I and II expression in the rat NIH-HS [41]. We measured the number of circulating CD4 and CD8 T cells and extracellular MHC class I and II expression by flow cytometry in more than 2000 HS rats, of which 1407 were genotyped at 265,551 SNPs. Each HS rat chromosome was reconstructed as a mosaic of the founder genomes, and QTLs were mapped using two methods that take the different levels of relatedness existing in the rat HS into account (see *Materials and Methods*). We report QTLs detected at a false discovery rate of 10%, which corresponds to a negative logP threshold of 4.2 for the three measures studied here.

**Figure 1:**
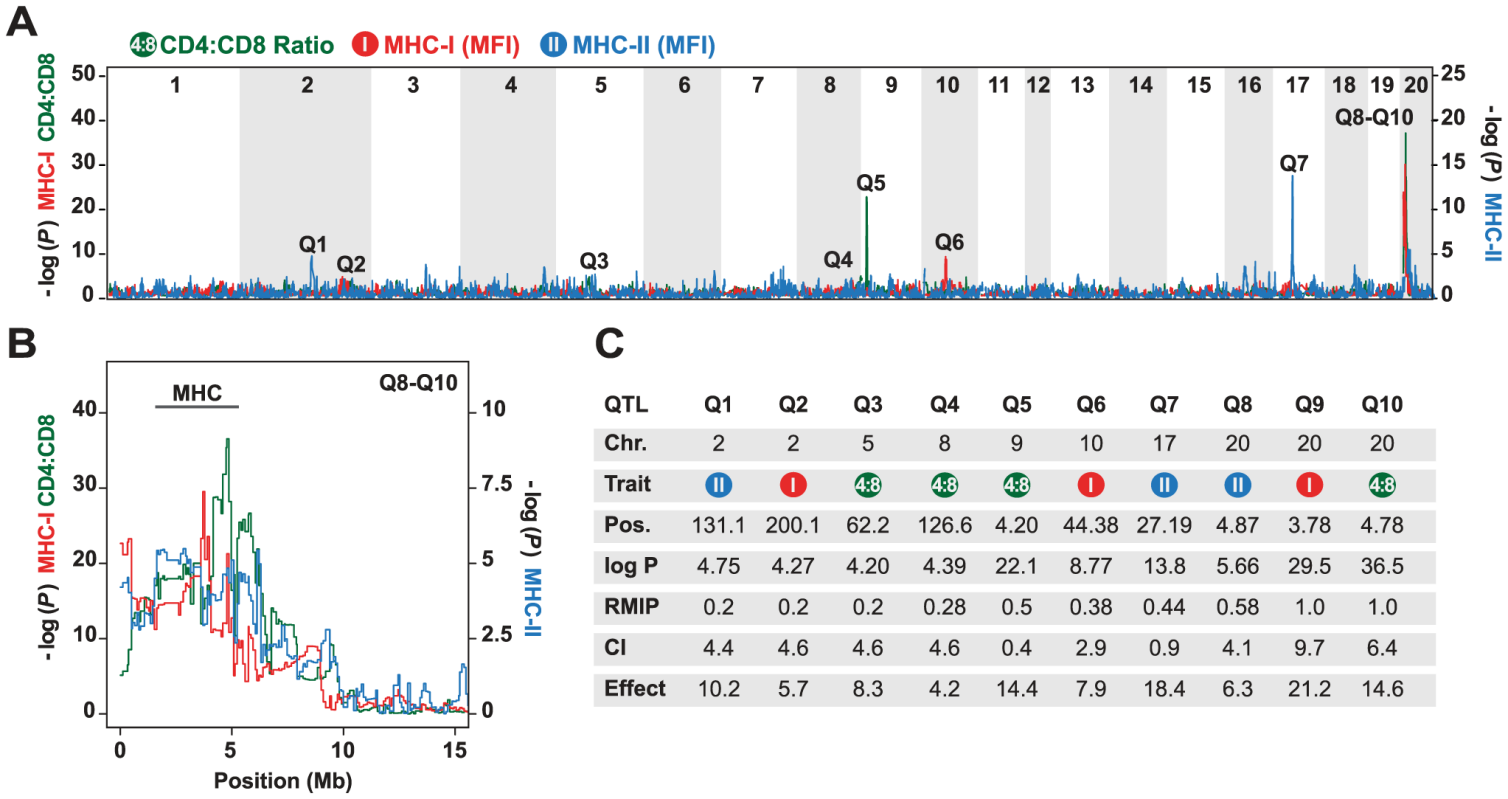
Genetic mapping in heterogeneous stock (HS) rats. (A) Genome scans of the CD4∶CD8 ratio (green), and the surface expression of MHC class I (MHC-I, red) and MHC class II (MHC-II, blue). The vertical axis shows the negative log *P* value for the 20 autosomes. Note that the left and right axis show −log *P* values for different phenotypes. Q1–Q10 indicate significant QTLs. (B) Close-up for Q8–Q10 on chromosome 20 showing the approximate location of the MHC region. (C) Position (in megabases, Mb) of peak marker (Pos.), confidence intervals (CI) in Mb, and effect sizes (%) of the peak markers in the QTLs shown in (A). The RMIP value is a measure of the probability that the loci are correctly identified (max = 1.0).

The mean ratio of circulating CD4 vs. CD8 T cells in the HS rats was 2.76 (range 1.0–13.8), which is concordant with data previously obtained in the inbred founder strains [42]. Variation in this phenotype was explained mainly by QTLs on chromosomes 9 and 20 (Fig. 1). The intervals most significantly associated with this phenotype were located at 4.20 Mb at the proximal end of chromosome 9 (−logP = 22.1), and at 4.78 Mb in the MHC region on chromosome 20 (−logP = 36.5). The effect sizes of these QTLs were estimated to be 14.4% and 14.6% respectively (upper bound; Fig. 1C). These QTLs also contributed to variation in absolute numbers of CD4 and CD8 T cells (data not shown, genome scans are accessible on the WTCHG website: http://mus.well.ox.ac.uk/gscandb/rat).

The surface expression of MHC class I was determined on granulocytes using a widely reactive MHC class I antibody to Ia and Ib molecules. QTLs for this phenotype were found on chromosome 2 (−logP = 4.27), 10 (−logP = 8.77) and 20 (−logP = 29.5), of which only the latter coincided with a QTL for CD4∶CD8 T cell ratio. Three regions were associated with MHC class II (RT1-B) expression on B cells. These were located on chromosome 2 (−logP = 4.75), 17 (−logP = 13.8) and 20 (−logP = 5.66). The most significantly associated marker on chromosome 20 was located at 4.87 Mb in the MHC region. Because the median 90% confidence interval for the position of the QTLs mapped in the rat HS is 4.5 Mb, this QTL overlapped with the QTL identified for CD4∶CD8 T cell ratio on this chromosome.

In summary, using a genome-wide approach, we showed that CD4∶CD8 T cell ratio and MHC expression might be regulated by a common QTL or a set of closely linked QTLs in the rat MHC region. However, the majority of identified QTLs for these traits did not co-localize, suggesting that variation in MHC expression is not a strong determinant of the CD4∶CD8 T cell ratio.

### Generation of MHC recombinant congenic strains

We next aimed to isolate the intervals associated with the phenotypic variation in the MHC, since the mapping in the HS rats did not distinguish whether a single gene with pleiotropic action or different MHC genes controlled MHC class I and II expression and the CD4∶CD8 T cell ratio.

**Figure 2:**
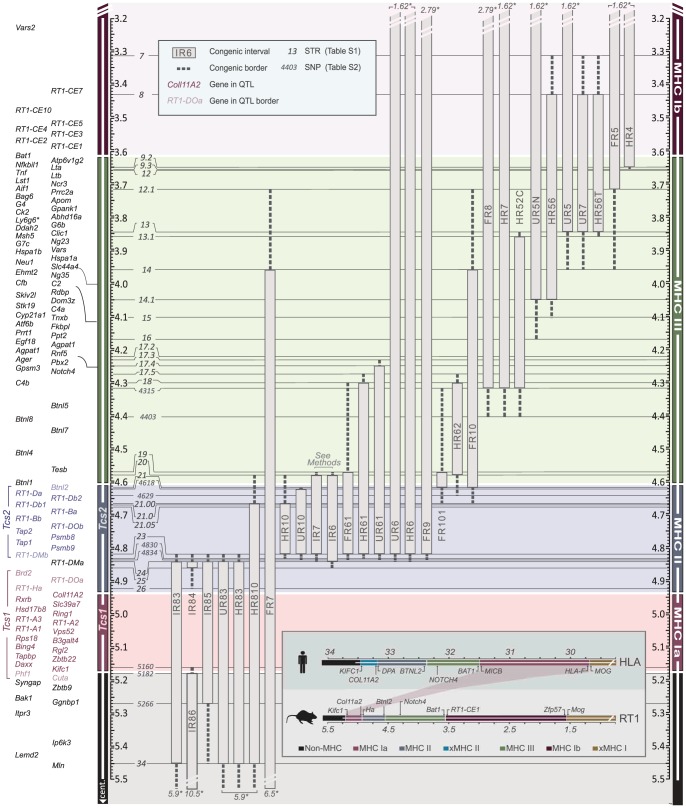
Physical map of the rat MHC region. The map was constructed according to the NCBI build 3.4 genome assembly. Genes (left) are depicted according to scale (positions in Mb), except for positions indicated with crotchets. The gross organization of MHC-Ia, II, III and Ib -regions are adopted from Hurt et al. [9]. Recombinant congenic strains are shown as gray vertical bars with dashed lines representing congenic borders (intervals of unknown genotype). Markers, short tandem repeats (STRs) and single nucleotide polymorphisms (SNPs), are shown in italic numbers. Numbers with asterisks at the top and bottom of the figure represent the position of the closest negative (DA) marker. Genes in *Tcs1* are red and in *Tcs2* blue (see box for definitions). Inset shows the organization of the human (HLA) and rat (RT1) MHC regions. Non-recombinant congenic strains, which have fragments spanning the entire MHC region, are not shown. xMHC-II, extended MHC class II region; xMHC-I, extended MHC class I region.

**Figure 3:**
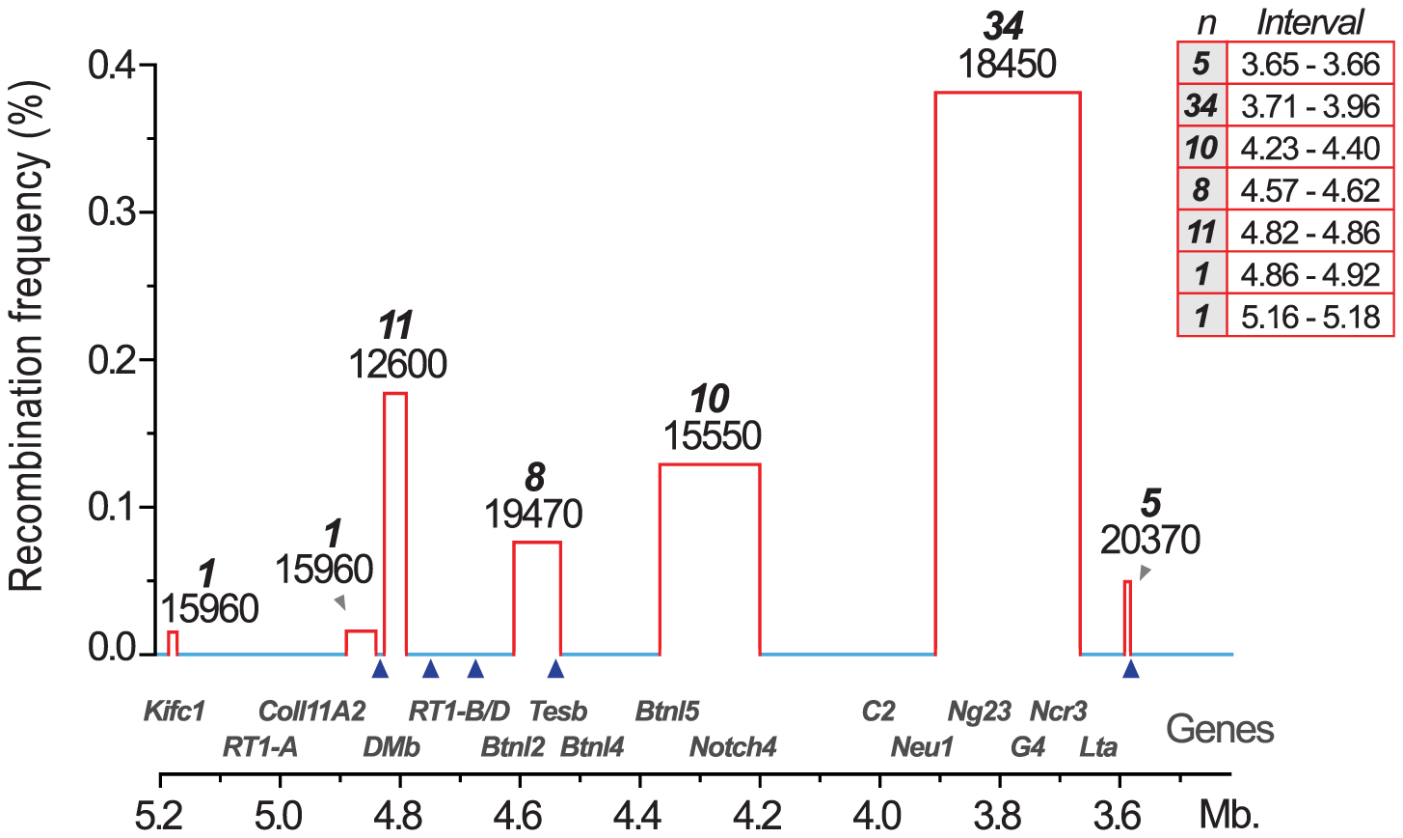
Recombination hotspots and haplotype blocks in the rat MHC. Recombination activity was assessed over 2(3.4–5.4 Mb). Blue lines represent cold regions (haplotype blocks) with low recombination activity. Regions with recombinations are depicted as red bars. The width of the bars, and the table inset (right), represent recombination intervals. Numbers above bars in bold face represent the observed numbers of recombinations within the interval with the number of analyzed meiotic events shown below. The height of the bars indicate individuals with recombinations (in %). Blue triangles represent recombination hotspots in humans and are adopted from Cullen et al. [6].

We therefore established MHC congenic strains with RT1^f^, RT1^i^, RT1^u^ and RT1^h^ haplotypes on the homozygous DA (RT1^av1^) background that showed phenotypic variation in CD4∶CD8 T cell ratio and class I and II expression (Table 1). We produced a panel of more than 40 RCS with segments in the MHC-I, -II and -III regions (selected strains are shown in Figure 2). We identified 70 recombinations within a 2 Mb genomic region (3.4–5.4 Mb) (Fig. 3) and mapped the recombination breakpoints using 67 SNP and short-tandem repeat (STR) markers (Table S1 and Table S2) to intervals of 2–270 kb. Three recombination hotspots were identified near *DMb*, *Btnl2* and *Lta* (Fig. 3), which are also located close to recombination hotspots in the human HLA [6], [8], [43], [44]. We did not identify any recombination events in the MHC-II region between *DMb* and *Btnl2*, despite analyzing ∼29,000 meiotic events. Extensive LD was also observed between *Kifc1* and *Coll11A2* in the MHC-I region where only a single recombination near the *Kifc1* gene was identified among the ∼16,000 analyzed meiotic events. Thus, recombinations in the RT1 between the MHC-I and -II regions occur relatively frequently (0.2%), while the recombination activity within each of these regions is extremely low. The extensive LD between *DMb* and *Btnl2* as well as between *Kifc1* and *Coll11A2* therefore gave rise to two haplotypes that could be isolated as congenic segments in the RCS panel.

**Table 1:** CD4∶CD8 T cell ratio and extracellular MHC class I and II expression in spleens of congenic and recombinant strains.

			RT1^a^						MHC-II^b^ *^,^* d	
	Strain	*n*	A	B	D	CEM	CD4∶CD8	MHC-I^b^ *^,^* c	RT1-B	RT1-D
WT	DA	10	*a*	*a*	*a*	*a*	4.1±0.3	100±12	100±5.4	100±7.4
PS	DA.1F	10	*f*	*f*	*f*	*f*	2.9±0.2^e^	83±7.0^f^	67±4.9^e^	98±6.6
PS	DA.1H	6	*h*	*h*	*h*	*h*	7.5±0.4^f^	92±4.3	74±4.8^e^	88±3.9^f^
PS	DA.1I	10	*i*	*i*	*i*	*a*	6.9±0.4^e^	67±5.4^e^	97±7.2	97±5.6
PS	DA.1U	7	*u*	*u*	*u*	*u*	2.4±0.3^e^	92±6.0	79±6.5^e^	96±5.4
RCS	DA.1FR9	7	*a*	*f*	*f*	*a*	2.9±0.1^e^	96±8.0	58±4.3^e^	101±16
RCS	DA.1HR10	6	*a*	*h*	*h*	*a*	3.8±0.3	75±7.0^f^	78±8.5^f^	85±5.3^f^
RCS	DA.1IR7	9	*a*	*i*	*i*	*a*	4.4±0.4	103±4.7	102±13	100±9.4
RCS	DA.1UR10	7	*a*	*u*	*u*	*a*	3.0±0.1^e^	79±3.8^f^	70±2.3^e^	107±8.0
RCS	DA.1HR83	5	*h*	*a*	*a*	*a*	7.1±0.6^f^	85±6.0^g^	101±11	98±7.1
RCS	DA.1IR85	5	*i*	*a*	*a*	*a*	8.2±0.7^f^	68±1.1^f^	104±0.9	101±3.6
RCS	DA.1UR83	5	*u*	*a*	*a*	*a*	3.2±0.2^f^	88±2.4^g^	103±4.5	100±4.1

a RT1 haplotype designations are based on genotyping data (see *Methods*);

b Strains were compared in separate experiments to DA littermate controls. The mean fluorescent intensity was then normalized to the expression of DA, which was given an arbitrary value of 100.

c MHC-I (class Ia and Ib; OX18) extracellular expression on WBCs.

d MHC class II expression on B cells.

Significant differences compared to DA:

e *P<0.001*;

f *P<0.01*;

g *P<0.05*.

WT, wildtype; PS, parental strain; RCS, recombinant congenic strain. Shown are mean values ±SD.

### Two linked QTLs in RT1 regulate CD4∶CD8 T cell ratio and MHC expression

The RCS panel allowed the identification of two intervals in RT1 associated with the variation in CD4∶CD8 T cell ratio and MHC expression (Table 1). The first interval, which we named *T cell selection QTL 1* (*Tcs1*), was identified within the MHC-I region (Fig. 2) using DA.1IR85, DA.1HR83 and DA.1UR83 (we refer to these strains as *Tcs1*-congenic strains). This QTL, which regulated class I cell surface expression and the CD4∶CD8 T cell ratio, was determined to 0.282 Mb (min-max: 0.242–0.323 Mb) based on the average size of the intervals of unknown genotype (congenic borders) in phenotype-negative DA.1IR86 and DA.1IR84 rats (Fig. 2). The second QTL, denoted *T cell selection QTL 2* (*Tcs2*), was mapped to the MHC-II region using the *Tcs2*-congenic strains DA.1UR10, DA.1HR10 and DA.1FR9 (Fig. 2). Since DA.1FR61 (Fig. 2) was not yet available at the time of the investigation, we used DA.1FR10 and DA.1FR8 (both phenotype-negative) to exclude genes in the MHC-III and *RT1-CEM* region of DA.1FR9 (Fig. 2). Recombination events in DA.1UR10 within *Btnl2* (between intron 5 and 6) and in DA.1HR10 within *RT1-DMb* (between intron 2 and 5) constituted the telomeric and centromeric boundaries of *Tcs2*, which was determined to 0.206 Mb (min-max: 0.197–0.214 Mb) (Fig. 2). This QTL regulated the cell surface expression of class I and II as well as the CD4∶CD8 T cell ratio.

*Tcs1* and *Tcs2* together explained all variation in the CD4∶CD8 T cell ratio and extracellular MHC expression associated with RT1 in the RCS. This suggests that these two linked haplotypes are also responsible for the phenotypic variation mapped to chromosome 20 in the HS. While *Tcs2* alone controlled MHC class II expression, both QTLs, *Tcs1* and *Tcs2*, controlled cell surface expression of MHC class I and the variation in CD4∶CD8 T cell ratio. The effect of the MHC-II region on MHC class I expression has previously been ascribed to allelic variants of *Tap2* [25]. Since the other genes in *Tcs2* are in strong LD with *Tap2*, we next compared the genetic variation of these genes in order to identify their individual contribution.

### Genes encoding proteins in the MHC class I and II pathways show variable degrees of sequence conservation

Two QTLs, *Tcs1* in the MHC-I region and *Tcs2* in the MHC-II region, were associated with the variation in CD4∶CD8 T cell ratio and MHC expression. In order to identify the causative genes in *Tcs1* and *Tcs2* associated with these traits, we determined the genetic variation of genes encoding proteins in the MHC class I and II pathways (all genes in *Tcs2, Tapbp* in *Tcs1*) by direct sequencing. These analyses of congenic (RT1^f,h,i,u^), wild-type (RT1^a^) and RefSeq (RT1^n^) haplotypes revealed 367 exonic SNPs (synonymous and nonsynonymous), 15 indels (insertions/deletions) and 168 amino acid (aa) substitutions in the corresponding proteins (Table 2).

**Table 2:** Sequence variants and allele distribution of genes in six rat MHC haplotypes.

*Gene* a	*Size* b	*SNP* c	*del* d	*Allele 1* e	*Allele 2*	*Allele 3*	*Allele 4*	*Allele 5*	*Allele 6*	*Subs* f	*Isof* g
*Btnl2* h	1542	4	0	*a n*	*u*					3	2
*RT1-Da*	768	7	0	*a f i h n*	*u*					1	2
*RT1-Db2*	861	2	3	*a f*	*i*	*n*	*u*			0	2
*RT1-Db1*	795	76	9	*a f i*	*h n*	*u*				35	3
*RT1-Ba*	768	66	3	*a i*	*h n*	*f*	*u*			40	4
*RT1-Bb*	792	72	0	*a i*	*h n*	*f*	*u*			38	4
*RT1-DOb* i	819	19	0	*a i*	*h n*	*f*	*u*			7	4
*Tap2*	2112	61	0	*a i*	*h n*	*f*	*u*			28	4
*Psmb8*	831	14	0	*a i*	*h n*	*f*	*u*			3	3
*Tap1*	2178	26	0	*a*	*f*	*i*	*h*	*n*	*u*	10	6
*Psmb9*	661	5	0	*n i*	*f u*	*a*	*h*			1	2
*RT1-DMb*	785	8	0	*n i*	*a*	*f*	*h*	*u*		1	2
*RT1-DMa*	783	5	0	*a f h*	*n i*	*u*				0	1
*Tapbp*	1563	2	0	*a f n*	*h i*	*u*				1	2

a All genes except *RT1-DMa* and *Tapbp* are encoded within the *Tcs2* locus.

b Size of the coding sequences (cds) in bp.

c Total number of synonymous and nonsynonymous SNPs in the cds.

d Total number of insertions/deletions on gene level.

e Allele distribution between MHC haplotypes; DA (RT1^a^), DA.1F (RT1^f^), DA.1I (RT1^i^), DA.1H (RT1^h^), DA.1U (RT1^u^). Sequence information for RT1^n^ was obtained from NCBI GenBank.

f Total number of amino acid substitutions.

g Number of alternative isoforms at the protein level.

h *Btnl2* was analyzed in only two strains.

I Sequence information for RT1-DOb refers to the full-length transcript of this gene (see Fig. S1 for alternative transcript).

**Figure 4:**
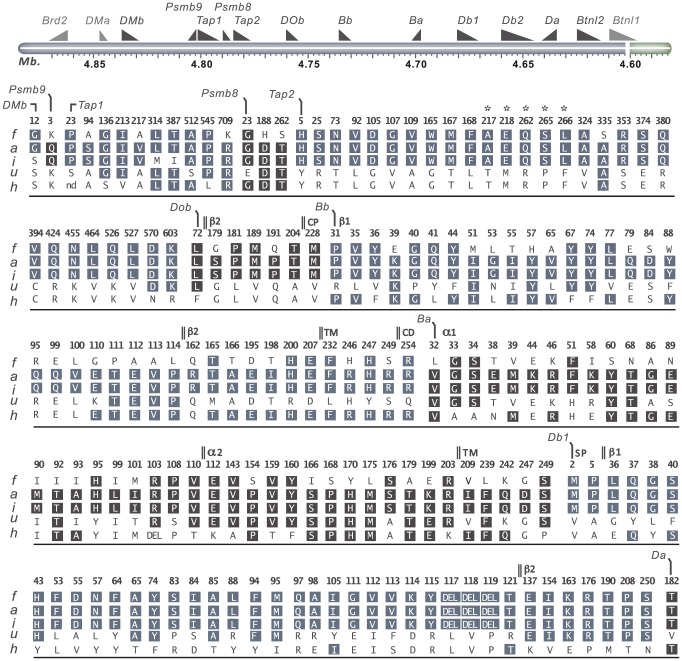
Coding variants in *T cell selection QTL 2* (*Tcs2*). Nonsynonymous- and structural variants in *Tcs2* were determined by Sanger sequencing of DA.1F (f), DA (a), DA.1I (i), DA.1U (u) and DA.1H (h) (see also Table 2). Amino acid substitutions are indicated in standard single letter codes and insertions/deletions as DEL. Gene annotations are from UniProt; protein domains depicted in *DOb* are derived from the human homolog. Letters in boxes depict residues with background allele (DA) with numbers above indicating amino acid positions in the translated cds. On top is a schematic illustration of the genes in the region according to the 3.4 genome assembly (genes outside the QTL are shown in gray). Annotations used: TM, transmembrane domain; CD, cytoplasmic domain; CP, connecting peptide; β1, beta 1 domain; β2, beta 2 domain; α1, alpha 1 domain; α2, alpha 2 domain. *Residues associated with class-I modification (cim).

The genes associated with the MHC class I pathway (*Tap1, Tap2, Psmb8, Psmb9 and Tapbp*) showed variable degrees of sequence conservation (Fig. 4). The most conserved gene was *Tapbp*, with only a single nonsynonymous SNP (nsSNP) coding for R165H in the corresponding protein (alternative allele in the RT1^u^ haplotype, Table 2). *Psmb9* contained one nsSNP in a region coding for the propeptide while three nsSNPs were found in *Psmb8* (Fig. 4), although not in positions predicted to influence immunoproteasome assembly or the chymotrypsin-like activity of the corresponding protein [45]. By contrast, both *Tap1* and *Tap2* showed extreme sequence diversity. We identified six allelic variants of *Tap1*, including unique alleles for the RT1^i^ and RT1^h^ haplotypes, and four allelic variants of *Tap2* (Fig. 4). Three of the *Tap2* alleles have not been sequenced previously: *Tap2^h^*, *Tap2^f^* and *Tap2^i^*, of which the latter was found to be identical to *Tap2^a^* (Table 2). Our sequence analysis confirms previous categorization of these alleles using restriction endonucleases [23] as *Tap2A* (RT1^f^ and RT1^i^) and *Tap2B* (RT1^h^), respectively. This categorization of *Tap2* alleles is based on nsSNPs coding for the amino acids 217, 218, 262, 265, 266 (asterisked in Fig. 4) in the Tap2 polypeptide, which determine the peptide specificity of the TAP complex [46], [47].

Variable sequence diversity was also observed for the genes in the MHC class II pathway (*DOb*, *DMa* and *DMb*). The *Tcs1* and *Tcs2* intervals excluded the *DMa* gene as well as the only nsSNP in *DMb* (corresponding to aa position 12) (Fig. 4). The *DOb* gene was found to be more polymorphic in the rat than previously shown for mice and humans [48], with a total of seven aa substitutions in the corresponding protein (Fig. 4). Two transcripts of *DOb*, which differed in length by 77 bp due to a deletion in exon 3, were identified in both congenic strains and DA (Fig. S1 and Fig. S2).

The rat expresses two classical MHC class II loci, *RT1-B* and *RT1-D*. Three MHC class II genes, *RT1-Ba*, *-Bb* and *-Db1* were highly polymorphic across the six haplotypes studied here, whereas *RT1-Da* was largely conserved with only a single nsSNP (Fig. 4). The existence of a second putative β-locus of *RT1-D* has been suggested [9]. Our data show that this gene, *RT1-Db2*, is expressed and that it is monomorphic at the protein level in DA and MHC-congenic strains (data not shown). We could further show by mass spectrometry that RT1-Db2 forms an αβ-heterodimer with RT1-Da and confirm at the protein level that RT1-Db2 has a 22 residue extended cytoplasmic tail, which does not exist in RT1-Db1 (Fig. S3).

Taken together, among the genes in the MHC class I pathway, only *Tap2* had an allele distribution that correlated with the variation in MHC class I expression (Fig. 4, Table 1). The effect of different *Tap2* alleles on MHC class I expression has previously been established [25], [29], and we therefore suggest *Tap2* as the only causative gene in *Tcs2* for this phenotype. In addition, these data exclude functional polymorphisms in *Tapbp* as responsible for the *Tcs1* QTL in MHC-I. Hence, the variations in MHC class I expression and the CD4∶CD8 T cell ratio associated with this QTL are likely to be due to polymorphisms in the *RT1-A* genes and/or the number of functional protein-coding *RT1-A* genes per MHC-I haplotype.

### Expression of class I proteins in *Tcs1*-congenic rats

The number of *RT1-A* genes in standard inbred rat strains varies from one in the RT1^lv1^ and RT1^a^ haplotypes to three in the RT1^n,o,d,m^ haplotypes [14], [18]. It has therefore been suggested that the number of *RT1-A* genes influences the selection of CD8 T cells [36]. The expression of *RT1-A* at the protein level has previously been characterized using allotypic antibodies [18], [49]–[52], while the phenotypes we mapped in the HS and RCS were determined using the widely reactive MHC class I antibody OX18, which does not discriminate between different isoforms of RT1-A. In order to assess the number of functional protein-coding *RT1-A* genes in DA.1IR85 and to confirm the expression of RT1-A^a^, A1^h^ and A2^h^, and A^u^ as the only class Ia molecules in DA and the *Tcs1*-congenic strains, we analyzed trypsin-digested cell lysates of IFN-γ stimulated splenocytes from these strains by mass spectrometry. The comparison of DA and the *Tcs1*-congenic strains also allowed the identification and discrimination of classical Ia and non-classical Ib proteins, since these strains encode different *RT1-A* genes but the same *RT1-CEM* genes.

We identified between 5 and 17 peptides (Mascot score >20) per strain that matched rat class Ia and Ib entries in public databases. In DA, 11 of 17 identified peptides matched the RT1-A^a^ molecule described by Rada et al. [53], while four peptides matched the UniProt entry HA11_RAT (Table 3, Fig. S4). HA11_RAT is the UniProt entry of the class Ib gene *RT1-EC2* (RGD), which was isolated from a DA cDNA library as *clone 3.6* [53] and predicted to be the rat homologue of mouse *H2-Q10* [54]. All four peptides identified in DA.1UR83 were derived from a single class Ia molecule (Table 3, Fig. S5), which is consistent with previous observations that the RT1^a^ and the RT1^u^ haplotypes express only one class Ia gene each (*RT1-A^a^* and *RT1-A^u^*, respectively) [53], [55]. In DA.1IR85 and DA.1HR83, 12 and 13 peptides, respectively, allowed the discrimination of two different RT1-A isoforms (A1 and A2) in each strain (Table 3, Fig. S6 and S7). In DA.1HR83 we also identified one peptide unique to clone 3.6 and two peptides that were shared between all identified class I molecules (Fig. S7). None of the analyzed samples contained peptides unique to an A3 isoform, which has been cloned in rats with haplotype RT1^o/d/m^ [18] and RT1^n^ [14].

**Table 3:** MHC class I proteins expressed in splenocytes from DA and *Tcs1*-congenic strains.

			F pocket^a^		
Strain	UniProt Entry	Gene	77	97	116
DA	HA12_RAT	*RT1-A* a	D	E	D
DA	HA11_RAT	*Clone 3.6*	S	R	H
DA.1UR83	Q31256_RAT	*RT1-A^u^*	N	V	D
DA.1HR83	Q9QYQ2_RAT	*RT1-A2^h^*	S	L	F
DA.1HR83	Q9QYQ3_RAT	*RT1-A1^h^*	D	L	Y
DA.1HR83	HA11_RAT	*Clone 3.6*	S	R	H
DA.1IR85	Q6MGB9_RAT	*RT1-A1^n^*	N	R	Y
DA.1IR85	Q6MGB8_RAT	*RT1-A2^n^*	D	R	D

a Amino acids in the F pocket, which discriminate TAP-A- from TAP-B-linked RT1-A molecules [24].

Taken together, our results show that DA.1IR85 and DA.1HR83 express two functional protein-coding *RT1-A* genes, *A1^n^*/*A2^n^* and *A1^h^*/*A2^h^*, respectively. We did not identify an A3 isoform in DA.1IR85, which is unexpected since the RT1-A region in DA.1IR85 is supposedly derived from the BN strain (RT1^n^) [56]. In DA and DA.1UR83 we only identified one RT1-A molecule per haplotype, which is in line with previous studies [53], [55].

### A novel type of class-I modification influences class I expression

Having established the number of protein-coding *RT1-A* genes in DA.1IR85 and confirmed the protein expression of RT1-A^a^, A1^h^ and A2^h^, and A^u^, we continued assessing the impact of TAP-A and TAP-B on the expression levels of MHC class I molecules in *Tcs1* and *Tcs2*-congenic strains.

Mapping in the RCS identified two adjacent QTLs for class I expression, *Tcs1* and *Tcs2*. *Tcs2* aligned with the principles of the *classical* cim phenomenon [25]. *Tcs1*, by contrast, was unexpected since the *RT1-A* genes in the *Tcs1*-congenic strains are associated with *Tap2A*, which encodes the promiscuous TAP-A transporter. We therefore hypothesized that this QTL was due to a novel type of class I modification in which the cell surface expression of TAP-B-linked RT1-A molecules (RT1-A^n^, RT1-A^u^ and RT1-A^h^) was reduced by the presence of TAP-A. We termed this TAP-A-mediated class-I modification *inverse cim*. To test this hypothesis, we analyzed the intra- and extracellular expression of class I in the *Tcs1*-congenic strains (*Tap2A*), DA (*Tap2A*) and in two non-recombinant parental strains, DA.1H and DA.1U (both *Tap2B*).

The expression of class I was determined using the OX18 antibody that binds both A1 and A2 isoforms [57]. However, since OX18 in addition recognizes class Ib molecules [58], which may differ in numbers between the non-recombinant strains and DA, we also used the class Ia specific antibody F16-4-4 [59]. This antibody, on the other hand, reacts with a polymorphic determinant (Fig. S8 and [52]) and was therefore only used to compare the class I expression between strains with the same *RT1-A* haplotypes.

**Figure 5:**
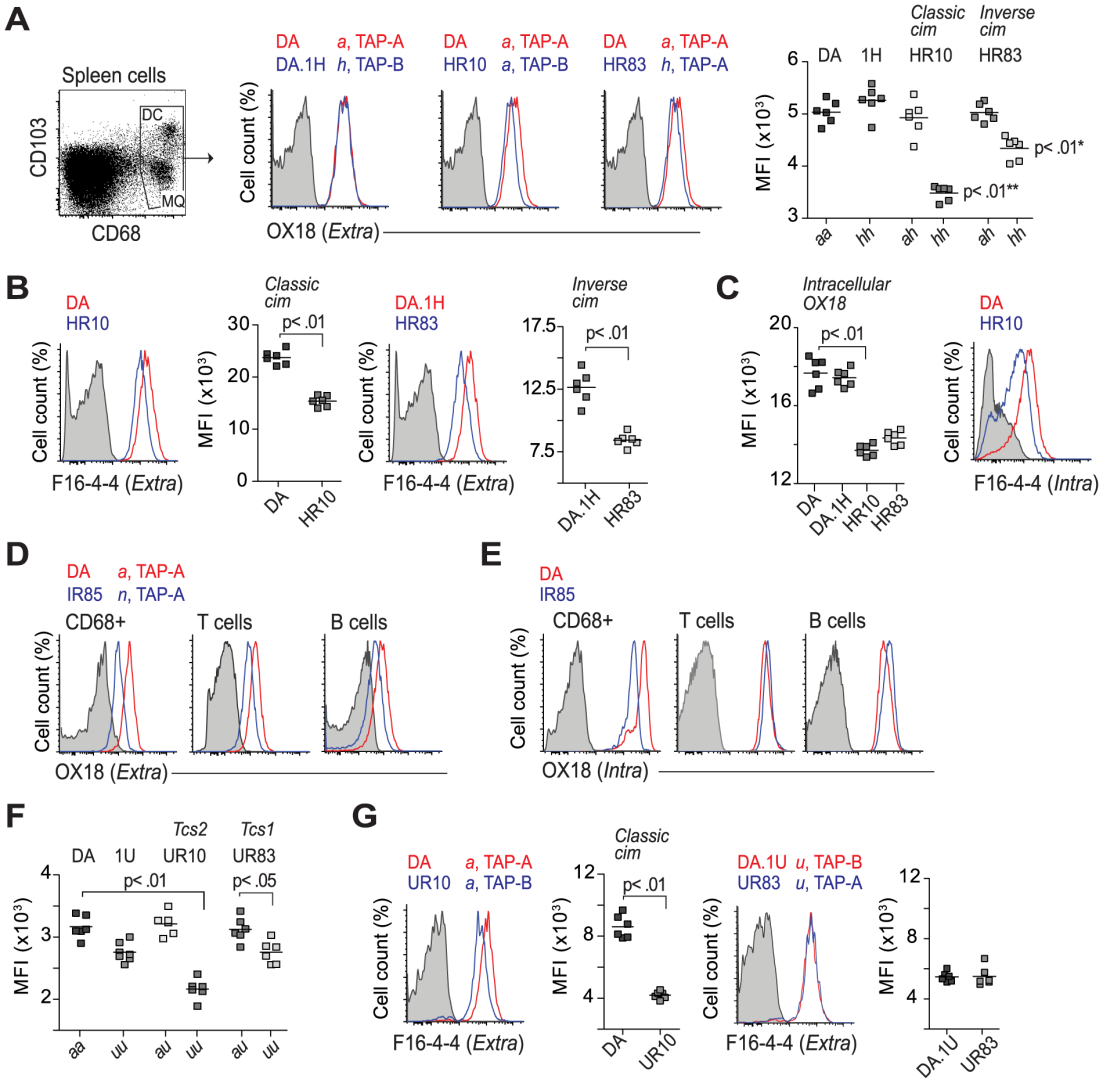
Regulation of class I expression by classical and inverse cim. (A) CD68+ cells were stained on the cell surface with OX18 (anti-class Ia and Ib). Histograms show representative samples from DA, DA.1H and DA.1H derived strains with different alleles of *RT1-A* and *Tap2* as stated on top. Data from all individuals are shown in scatterplot (far right); * significant compared to DA.1H (1H); ** significant compared to DA. (B) CD68+ cells stained with a class Ia specific antibody (F16-4-4). (C) T cells from animals shown in (A) stained intracellularly with OX18 (scatterplot) and F16-4-4 (histogram). (D–E) Subsets of leukocytes from DA and DA.1IR85 spleen stained extracellularly (D) and intracellularly (E) with OX18. Data are representative of 6 individuals per group. (F) Surface expression of MHC class I (OX18) on CD68+ cells from DA and DA.1U congenic strains. (G) CD68+ cells (same as in F) stained with F16-4-4. Vertical lines in scatterplots show mean values. Representative results of at least two independent experiments are shown.

We first analyzed the extracellular levels of class I on spleen cells expressing CD68, a marker of macrophages and dendritic cells in the rat [60]. Consistent with the leukocyte data shown in Table 1, CD68+ cells in DA.1HR83 and DA.1HR10 showed reduced surface levels of class I compared to DA and DA.1H when stained with OX18 (Fig. 5A) as well as with F16-4-4 (Fig. 5B). The lower levels of class Ia in DA.1HR83 (RT1-A^h^, TAP-A) compared to DA.1H (RT1-A^h^, TAP-B) suggest that RT1-A^h^ requires TAP-B for optimal export to the cell surface, which would be consistent with the inverse cim phenomenon.

The association of RT1-A^a^ to TAP-B has been shown to cause a relative retention of class I molecules in the ER [25], [29], implying that the level of class I should be higher intracellularly in DA.1HR10, and possibly also in DA.1HR83. However, we found that the intracellular levels of class I in these strains were also reduced compared to DA and DA.1H (Fig. 5C). These observations were also confirmed using F16-4-4 in DA.1HR10 (Fig. 5C), suggesting that both classical and inverse cim reduce the class I expression on the cell surface as well as inside the cell. DA.1IR85 showed extremely low extracellular levels of class I staining on all analyzed subsets of leukocytes as well as intracellularly in CD68+ cells (*P*<0.01; Fig. 5D, E). The intracellular levels of class I in lymphocytes (T and B cells), by contrast, did not differ between DA and DA.1IR85 (Fig. 5E), suggesting that at least one of the two RT1-A molecules in DA.1IR85 is not efficiently transported to the cell surface. It is difficult to fully evaluate the TAP restriction of RT1-A^n^ in DA.1IR85 since the parental strain, DA.1I, is itself a recombinant strain that expresses the *Tap2A* allele (Fig. 4). However, the class Ia genes in DA.1IR85, *RT1-A1^n^* and *RT1-A2^n^*, are naturally associated with *Tap2B* in BN, which does not show reduced class I expression compared to ACI (RT1-A^a^) [42]. This suggests that TAP-A is not an optimal transporter of peptides for RT1-A1^n^ and/or RT1-A2^n^ and that these molecules in DA.1IR85, as well as in DA.1I, are affected by the inverse cim.

As expected, the class I expression in DA.1IR7 and DA.1FR9 (RT1-A^a^, TAP-A), did not differ from DA (Table 1). Moreover, the *Tap2B* allele in DA.1UR10 has previously been shown to encode an inefficient transporter of peptides for RT1-A^a^ [61], which explains the low surface expression of class I in this strain (Fig. 5F,G). Leukocytes from the parental congenic strain DA.1U showed a trend towards lower levels of class I on the cell surface compared to DA (Table 1). This reduction in class I expression was statistically significant on CD68+ cells in DA.1U as well as in DA.1UR83 (Fig. 5F). Hence, the reduced expression of class I in DA.1UR83, which did not differ significantly from DA.1U (Fig. 5F,G), cannot be ascribed to polymorphisms in the *Tap2* gene and suggest that the expression of *RT1-A^u^* is regulated at the transcriptional level.

Our data support a novel type of cim in which RT1-A^h^ and probably also RT1-A^n^ require TAP-B for optimal expression at the cell surface. However, we also found evidence for a cim-independent regulation of extracellular class I levels for RT1-A^u^, suggesting that regulation also takes place at the gene level.

### Weak correlation between transcription and extracellular expression of class I

The variation in MHC class I protein expression between DA.1H and DA.1HR83 revealed that RT1-A1^h^ and/or A2^h^ were reduced in the context of TAP-A. However, whether the expression of MHC class I proteins in addition was influenced by a variation in *RT1-A* gene expression in the *Tcs1* congenic strain was still unclear.

**Figure 6:**
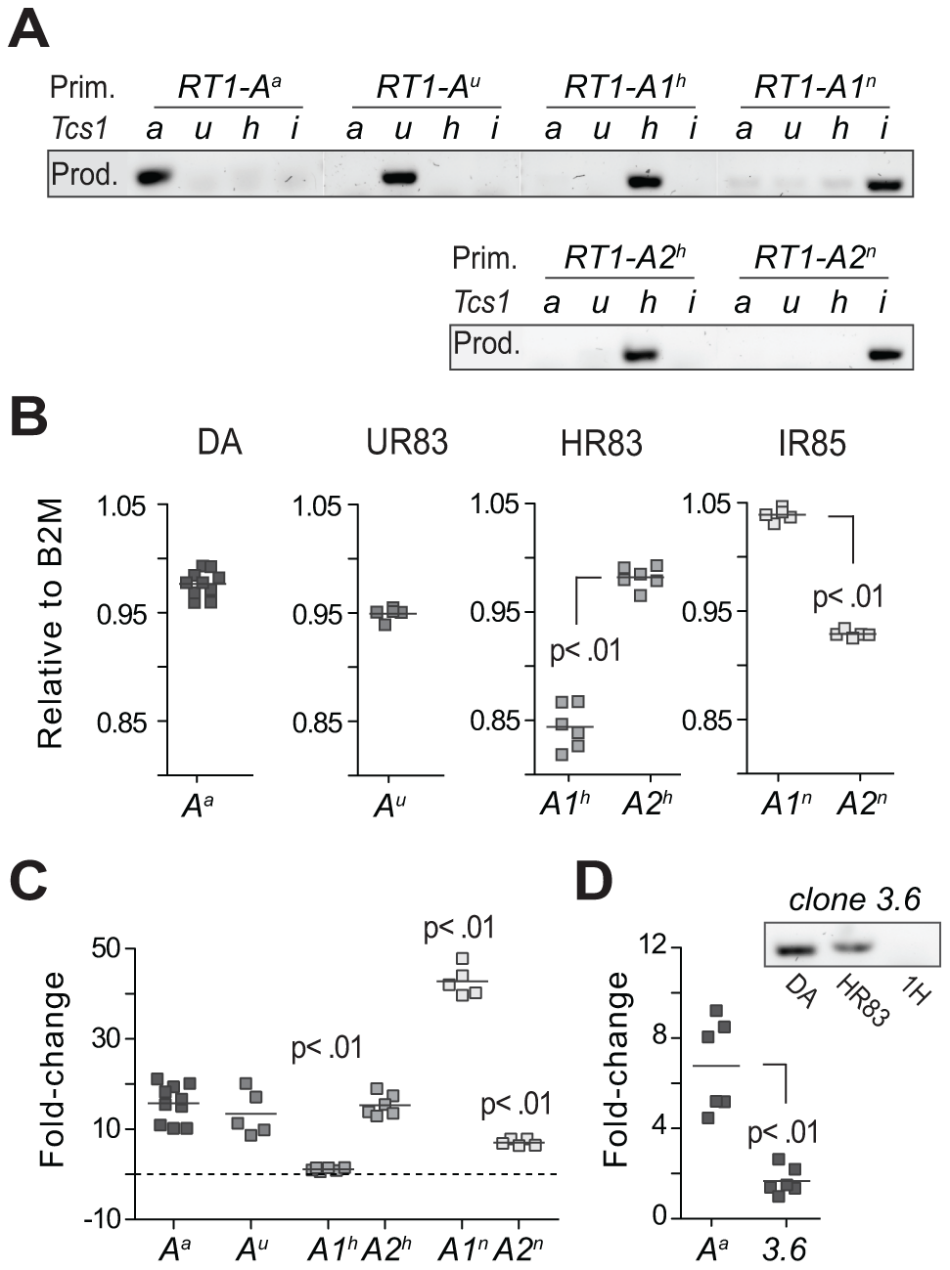
Transcriptional regulation of MHC class I genes. (A) Allele-specific primers (Prim.) used for quantitative RT-PCR showed minimal amplification of other RT1-A alleles and were therefore considered class Ia-specific (i.e. not cross-reacting to class Ib genes); PCR products (Prod.) are shown for DA (*a*), DA.1UR83 (*u*), DA.1HR83 (*h*) and DA.1IR85 (*i*). (B) Expression in spleen of RT1-A genes in DA and *Tcs1*-congenic strains relative to the expression of beta-2-microglobuline (*B2M*). (C) Variation (fold-change) in RT1-A gene expression between different congenic strains. Data show the mean expression of 2–4 different primer sets per target gene after normalization to 3 reference genes (Table S3). Significant differences compared to *RT1-A^a^*. (D) Expression of *RT1-A^a^* (class Ia) and *clone 3.6* (class Ib) in spleen from DA rats. The amplification of a product in DA.1HR83 but not in DA.1H (1H) indicates that the primers for *clone 3.6* are not cross-reacting to the *RT1-A^a^* gene in DA (adjacent figure).

We therefore determined the expression levels in the spleen of the six protein-coding *RT1-A* genes shown in Table 3. We designed allele-specific primers based on published *RT1-A* sequences to avoid off-target amplification of *RT1-CEM* genes (Fig. 6A). Each target was amplified with 2–4 different primer sets and the levels of product were averaged on the gene level and compared to the expression of beta-2-microglobulin (*B2M*, Fig. 6B) and to three reference genes (Fig. 6C). We observed the highest transcript levels for *RT1-A1^n^* in DA.1IR85, which was ∼5-fold higher than *RT1-A2^n^* in this strain and ∼3-fold higher than *RT1-A^a^* in DA. Hence, the high levels of intracellular MHC class I proteins in DA.1IR85 T and B cells (Fig. 5E) correlated with a high expression of *RT1-A1^n^* on the gene level. By contrast, such correlation could not be found for *RT1-A2^h^* in DA.1HR83 and *RT1-A^u^* in DA.1UR83, which had comparable expression levels to *RT1-A^a^* in DA despite their significant reduction in protein expression (Fig. 5).

We also compared the expression of class Ia and Ib genes in DA and found ∼6-fold higher levels of *RT1-A^a^* compared to *clone 3.6* (Fig. 6D), which was the only class Ib gene that was identified at the protein level (Table 3). Finally, we assessed the impact of *Tcs1* on genome-wide transcription by exon-microarray. This showed that all genes that were differentially expressed between DA and DA.1IR83 (which has a slightly larger congenic fragment compared to DA.1IR85) were located within the *Tcs1* region (Fig. S9). Hence, the phenotypic variation associated with *Tcs1* is unlikely to be due to trans-acting factors outside of the QTL.

Taken together, the extracellular expression of class I proteins correlated poorly with the expression at the gene level. These data therefore support the conclusion that class I expression on the cell surface is largely regulated by polymorphisms affecting the peptide binding pocket of the RT1-A molecules and the TAP transporter.

### Regulation of MHC expression by dendritic cells is similar in the thymus and spleen

The genetic variation in *Tap2* influenced the extracellular expression of class I molecules in the spleen. Next, we determined the expression of MHC on thymic DCs, which control the egress of T cells by eliminating self-reactive cells during negative selection [62].

**Figure 7:**
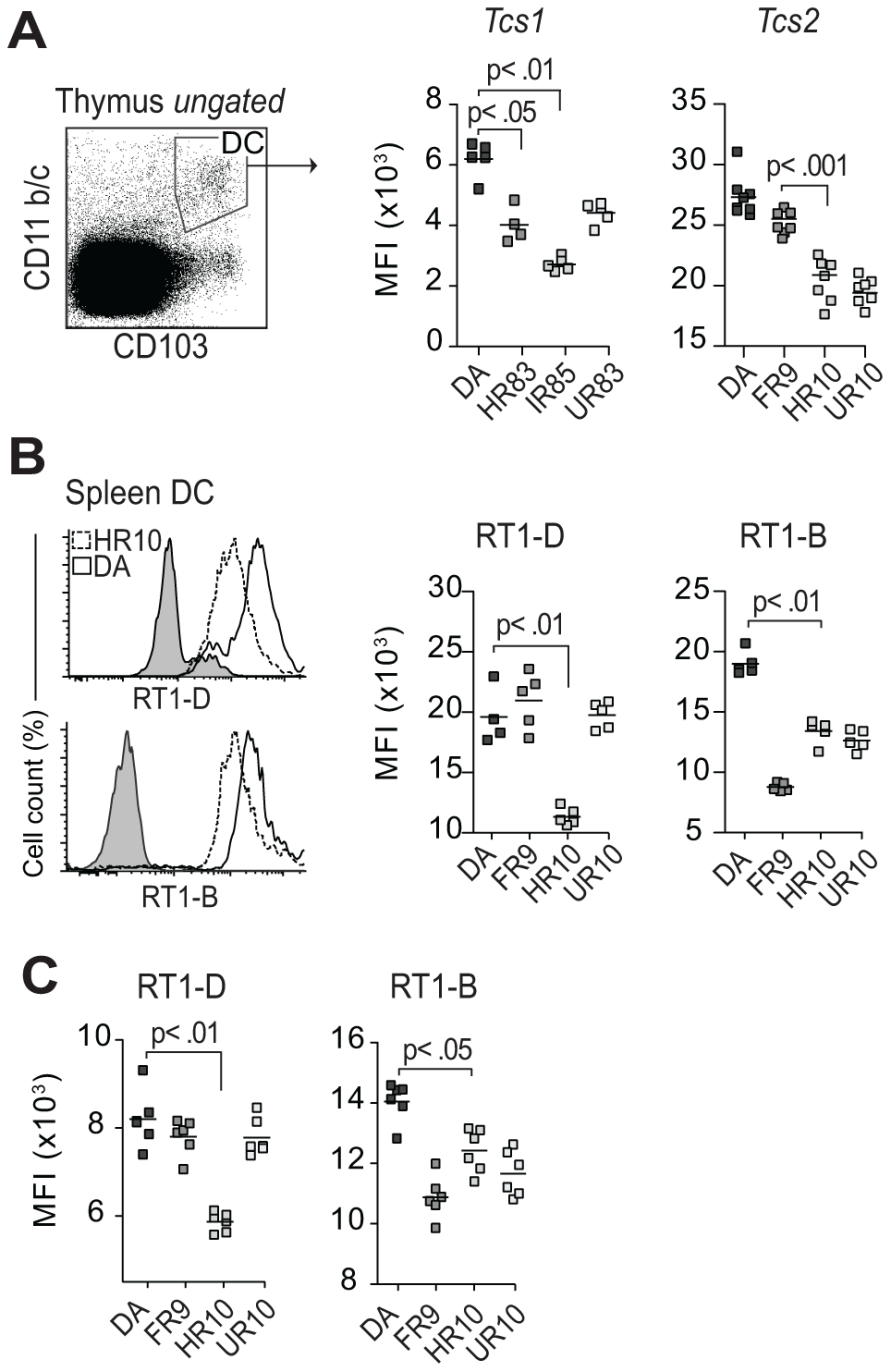
*Tcs1* and *Tcs2*-congenic strains show similar variation in extracellular MHC expression in thymus and spleen. (A) Thymic conventional DCs (CD103+, CD11b/c+) stained extracellularly for class I (OX18). Scatterplots show results from two different experiments with *Tcs1* and *Tcs2*-congenic strains. The variation between the strains is comparable to data shown in Figure 5 for CD68+ cells. (B–C) Splenic DCs (CD103+, CD68+, CD11b/c+) (B) and thymic DCs (C) stained extracellularly for RT1-D (OX17) and RT1-B (OX6). Histograms show representative samples from DA (solid lines) and DA.1HR10 (dashed lines).

Comparing the class I expression at the protein level between thymic DCs (Fig. 7A) and CD68+ cells in the spleen (Fig. 5) showed that the variation between the strains was similar in both organs. This suggests that cim affects a broad repertoire of cells in different tissues. Likewise, the variation in class II expression on DCs in *Tcs2*-congenic strains was similar in the spleen (Fig. 7B) and in the thymus (Fig. 7C), as well as between DCs and B cells in the spleen (Table 1). Thus, we established that the genetic variation in *Tap2* also affects the extracellular class I expression on antigen presenting cells in the thymus. We next aimed to determine if *Tap2* had a pleiotropic effect and was also responsible for the variation in CD4∶CD8 lineage commitment.

### Classical cim affects lineage commitment and negative selection of CD8 T cells

Classical and inverse cim influenced class I expression on thymic DCs. We hypothesized that the changes associated with cim in class I expression and/or in the quality of the peptide repertoire would affect thymic selection and thereby contribute to the variation in peripheral CD4 and CD8 T cell numbers.

**Figure 8:**
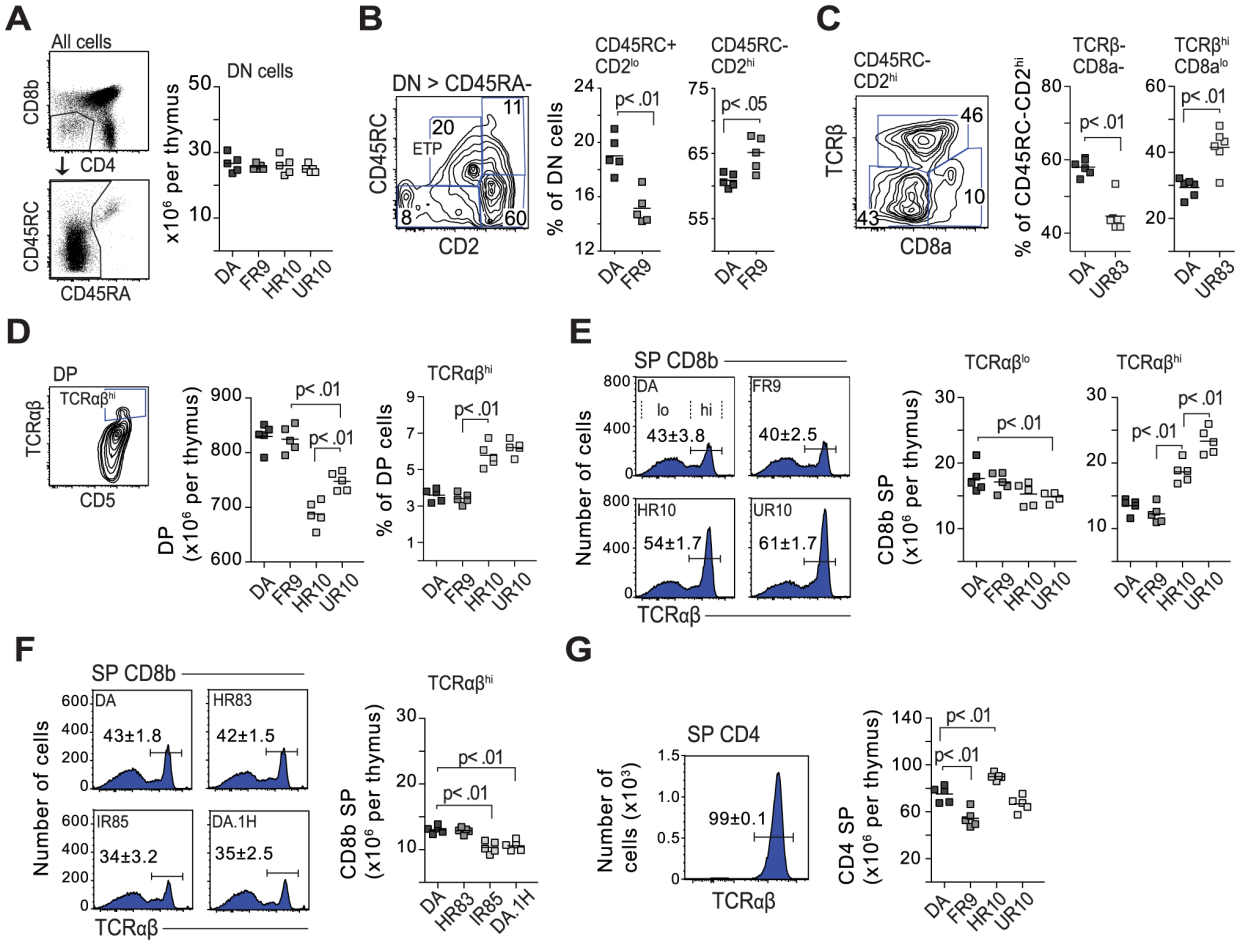
Class-I modification reduces negative selection of CD8 cells. (A) The QTLs in *Tcs1* and *Tcs2* did not affect the total number of double negative cells (DN; CD4−, CD8b−). B cells (CD45RA+) were excluded from the DN gate. (B) DN cell maturation is defined by CD45RC and CD2 (see Fig. S10). DN cells in DA.1FR9 showed a lower frequency of early thymic precursors (ETP) compared to DA. Counter plot shows gating strategy with numbers indicating percent (%) of parent population (stated above plot). (C) Counter plot shows CD45RC−, CD2^hi^ DN cells from DA.1UR83, and scatter plots the frequency of TCRβ− and TCRβ+ (DN4 in mouse) cells in DA and DA.1UR83. (D) Thymi from *Tcs2*-congenic strains with TAP-B (HR10 and UR10) contained fewer double positive (DP) cells but more cells (in %) with high TCR expression. (E) TAP-B strains (HR10 and UR10) showed higher frequencies (histograms) and total numbers (scatter plots) of CD8 single positive (SP) cells with high TCR expression. Numbers (%) in histograms represent mean-values ±SD of cells with high TCR expression (gated, *n* = 5). (F) Frequencies (histograms; *n* = 5) and total numbers (scatter plot) of CD8SP cells with high TCR expression in DA.1H (RT1-A^h^, TAP-B) and in strains with low levels of surface MHC class I (HR83 [RT1-A^h^, TAP-A] and IR85 [RT1-A^n^, TAP-A]). (G) Virtually all CD4SP cells express high levels of TCR. Histogram shows expression of TCR on CD4SP thymocytes in DA (*n* = 5). Scatterplot shows total number of CD4SP cells per thymus in *Tcs2*-congenic strains.

The different maturation stages of thymocytes are defined by the expression of various surface markers, which differ between rats, mice and humans [63], [64]. In the rat, double negative (DN) cells are defined according to the expression of CD45RC and CD2 [65] (see also Figure S10). We first determined the progression from the DN to the double positive (DP) stage in 6.5 week-old *Tcs1*- and *Tcs2*-congenic rats. We found no variation in the total number of DN cells (Fig. 8A, shown for *Tcs2*-congenic strains), while the frequency of early thymic precursors (CD45RC+, CD2^lo^) was reduced in DA.1FR9 compared to all other strains (Fig. 8B, shown for DA vs. DA.1FR9). Moreover, all strains showed similar frequencies of immature CD8α single positive (ISP) cells (data not shown), whereas DA.1UR83 showed a greater proportion of cells at late DN stage (corresponding to DN4 in the mouse), in which all cells express TCRβ on the cell surface (Fig. 8C, shown for DA vs. DA.1UR83).

Positive selection takes place in the thymic cortex where DP cells expressing intermediate levels of TCR interact with MHC expressing stromal cells. DP cells that express a functional TCRα chain, which can pair with TCRβ and recognize self-MHC, are positively selected. We determined the total number of DP cells in the thymus and found lower numbers in the strains expressing RT1-A^a^ and TAP-B (DA.1HR10, DA.1UR10) (Fig. 8D). In addition, DP cells in these strains showed a greater proportion of mature cells, which express high levels of TCR and CD5 (Fig. 8D) [64]. Such a reduction of DP cells was not observed in other strains with low expression of class I, such as DA.1IR85 (RT1-A^n^, TAP-A) (data not shown), suggesting that it is not the lower expression of class I that influences the number of DP cells in DA.1HR10 and DA.1UR10 but rather the quality of the class-I peptide repertoire.

Next, we determined how genes in the MHC-I and II regions influence lineage commitment and negative selection. TCR cross-linking of DP cells *in vitro* has been shown to generate CD8SP cells in the rat and CD4SP cells in the mouse [66], [67]. As shown in Figure 8E, ∼60% of CD8SP cells in DA express low or intermediate levels of TCR, which is in marked contrast to CD8SP cells in the mouse where essentially all are TCR^hi^ [68]. However, both the frequency and the total number of CD8SP TCR^hi^ cells varied substantially among the strains: the majority of CD8SP cells in strains with TAP-A expressed low TCR levels whereas most CD8SP cells in DA.1HR10 and DA.1UR10 (TAP-B) were TCR^hi^ (Fig. 8E and 8F). A similar increase in TCR^hi^ expressing CD8SP cells was not observed in DA.1H (RT1-A^h^, TAP-B) (Fig. 8F), indicating that the increase of CD8SP cells in DA.1HR10 (RT1-A^a^, TAP-B) is cim-dependent. Neither was it observed in *Tcs1*-congenic strains with low surface expression of class I (DA.1HR83, DA.1IR85; Fig. 8F), which suggests that it is the quality of the peptide repertoire and not the lower expression of class I that is responsible for the increase of CD8SP TCR^hi^ cells in DA.1HR10 and DA.1UR10.

In contrast to CD8SP cells, virtually all CD4SP cells were TCR^hi^ (Fig. 8G). All *Tcs1*-congenic strains had similar numbers of CD4SP cells (data not shown), while we observed an increase of total CD4SP cells in DA.1HR10 and a decrease in the same subset in DA.1FR9 compared to DA and DA.1UR10 (Fig. 8G). The variation in the number of CD4SP cells probably reflects the genetic variation in the classical MHC class II genes, in particular in the *RT1-B* genes since the *RT1-D* genes are conserved between DA and DA.1FR9 (Fig. 4). It is further unlikely that the variation in CD4SP cells is cim-dependent since only DA.1HR10, and not DA.1UR10, showed an increase in CD4SP cells.

These data suggest that classical cim reduces negative selection of class I restricted thymocytes, probably by limiting the complexity of available peptides in the ER. In addition, cim may influence positive selection as shown by the reduced number of DP cells in DA.1HR10 and DA.1UR10.

### Classical cim influences the number of peripheral CD8 T cells

**Figure 9:**
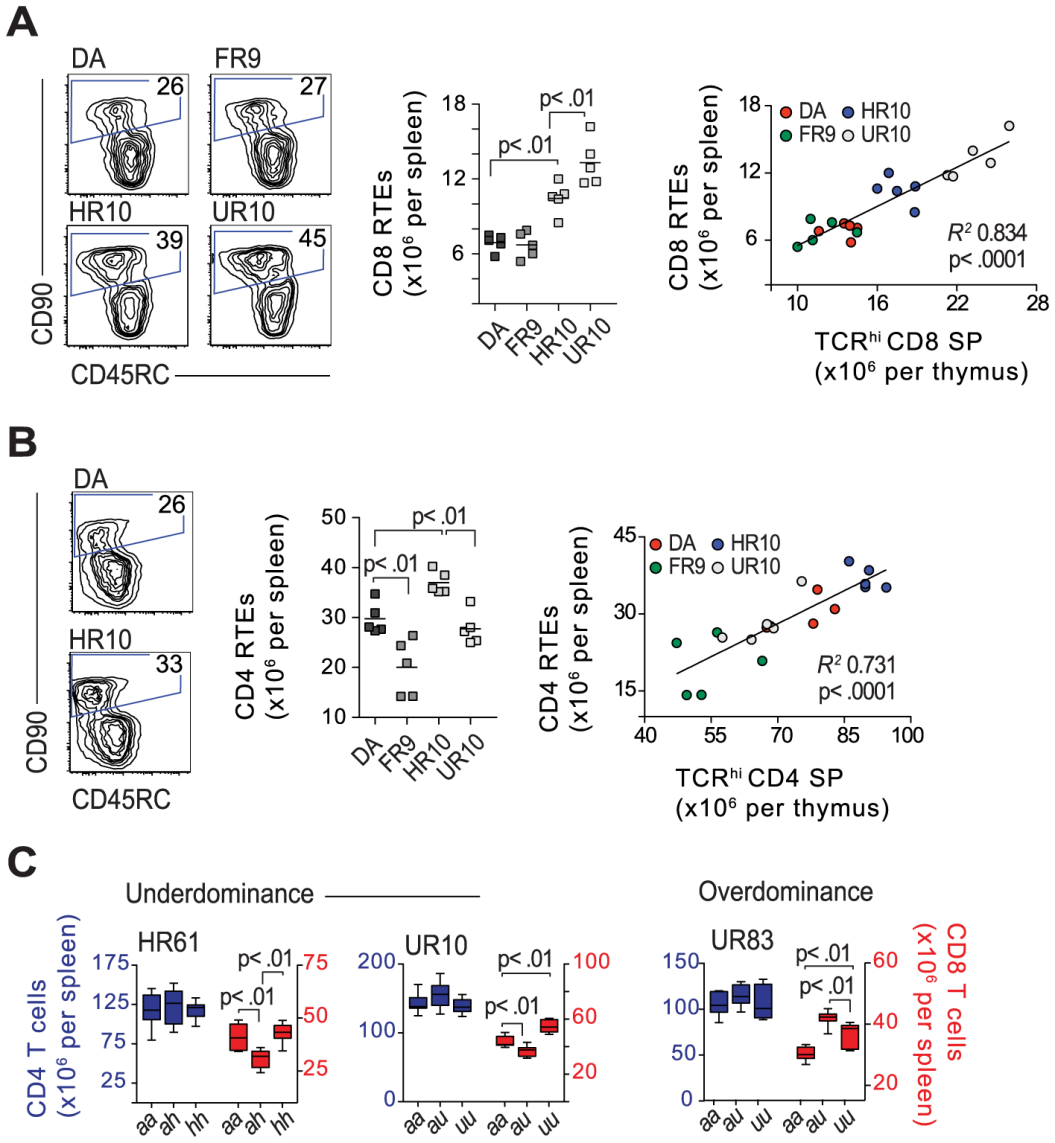
Classical cim influences the number of CD8 recent thymic emigrants. (A) Recent thymic emigrants (RTEs) express CD90 and low levels of CD45RC. Counterplots show CD8 RTEs (gated) in the spleen of 6.5-week old congenic and DA rats (representative samples, numbers in gates show percent of CD8 T cells). Scatterplot shows total number of CD8 RTEs per spleen, which correlates to the absolute number of CD8SP TCR^hi^ cells in the thymi from the same animals (far right). (B) The corresponding staining (as shown in A) for CD4 RTEs, including total numbers (scatterplot) and correlation to CD4SP TCR^hi^ cells in the thymus. (C) Absolute numbers of CD4 (blue) and CD8 (red) T cells in the spleen of 13-week old rats. Strains and genotypes are shown above and below graphs, respectively; *n* = 5–7 per group.

Class-I modification was associated with an increased number of CD8SP TCR^hi^ cells in the thymus. However, it was unclear whether this also led to an increase of CD8 T cells in the periphery. Thus, we assessed the number of CD8 recent thymic emigrants (RTEs), which in the rat can be distinguished by their expression of CD90 (Thy-1) and CD45RC [69], [70], in the spleen of 6.5 week-old *Tcs2*-congenic rats. The total number of CD8 RTEs was found to be significantly increased in DA.1HR10 and DA.1UR10 (RT1-A^a^, TAP-B) compared to DA and DA.1FR9 (RT1-A^a^, TAP-A). Furthermore, the number of CD8 RTEs in the spleen correlated significantly with the number of CD8SP TCR^hi^ cells in the thymus (*R^2^* = 0.83; *P*<0.001), indicating that CD8SP TCR^hi^ cells constitute a mature subset of thymocytes. CD8SP cells with low or intermediate TCR expression, by contrast, showed a weak inverse correlation with CD8 RTEs in the spleen (data not shown). Similarly to the CD8 RTEs, the CD4 RTEs in the spleen strongly correlated with CD4SP cells in the thymus (Fig. 9B). Differences in CD4 and CD8 RTEs were less pronounced in older rats (13–14 weeks of age), which also showed less, and slightly different, variations in total CD4 and CD8 T cell numbers compared to younger rats (Fig. S11). This emphasizes that other mechanisms, such as clonal expansion, may be more important than thymic output for maintaining the proportions of CD4 and CD8 T cells in older rats.

We further compared CD8 T cell numbers between heterozygous and homozygous DA.1HR61 (Fig. 2) and DA.1UR10 rats (both *Tap2B*). Since the *Tap2B* allele is recessive [29], it was expected that rats with heterozygous *Tap2* alleles had fewer CD8 T cells in the spleen compared to their *Tap2B* homozygous littermates (Fig. 9C). However, they had also fewer CD8 T cells than their DA littermates, a phenomenon known as underdominance, which has not previously been reported for T cell selection. The impact of heterozygosity was the opposite in DA.1UR83 (*Tap2A*), in which heterozygous rats had more CD8 T cells than their homozygous littermates (so called overdominance) (Fig. 9C). Hence, these phenomena were not directly related to cim but seem to be specific for the CD8 lineage, and may reflect the default CD8 lineage choice that has been reported in the rat [66], [67].

Taken together, we show that cim reduces negative selection of CD8 cells, increases the thymic output of CD8 T cells and thereby significantly contributes to the peripheral CD8 T cell repertoire.

## Discussion

We show that two linked haplotypes in the MHC-I and II regions control MHC expression and T cell selection in rats. A recombination between these haplotypes, which broke the evolutionary conserved linkage between *RT1-A* and *Tap2*, affected negative selection and lineage commitment of CD8 cells. This effect was found to be associated with the *Tap2B* allele and dependent on the co-expression of *RT1-A^a^*. The same combination of alleles has previously been shown to reduce the expression and alter the antigenicity of the RT1-A^a^ molecule, a phenomenon known as class-I modification (cim) [61]. We found in addition that the combination *RT1-A^h^* and *Tap2A*, encoding for the promiscuous TAP-A transporter, also reduced the extracellular expression of class I, which we term *inverse* cim. Thus, our results show how natural polymorphisms in *Tap2* modify class I expression and alter T cell selection in rats.

Following the identification of *Tap2* as a trans-acting modifier of RT1-A^a^ antigenicity [25], it has been thoroughly investigated how different TAP transporters are associated with changes in RT1-A protein expression and the RT1-A peptide repertoire. These studies suggest that TAP-A and TAP-B are equally efficient in transporting peptides with hydrophobic C-terminal residues, while TAP-B has weak affinity for peptides with basic C-terminal residues [18], [22], [26]–[28]. These differences were exploited here to assess how an altered spectrum of class I peptides influences T cell selection in animals with natural variations in the TCR and MHC loci.

Thymic DCs in the corticomedullary junction present antigens to positively selected thymocytes ([71], reviewed in [72]). Thymocytes with high affinity for self-peptide:MHC complexes are eliminated through negative selection. However, if the peptide repertoire on the DCs is limited, as in DA.1HR10 and DA.1UR10 (RT1-A^a^, TAP-B), fewer thymocytes will express TCRs with high affinity for peptide:MHC complexes and fewer cells will therefore undergo negative selection. We showed in addition that less negative selection in rats with RT1-A^a^ and TAP-B allotypes correlated with an increased number of CD8 RTEs in the periphery. Thus, it seems possible that cim may lead to an escape of autoreactive CD8 T cells. Preliminary results from our laboratory, however, show that CD8 cells from DA.1HR10 and DA.1UR10 produce lower levels of pro-inflammatory cytokines compared to strains expressing RT1-A^a^ and TAP-A (Tuncel and Haag, unpublished data).

Although the major effect of cim appears to be during negative selection, it also influenced the number of DP cells. This finding is consistent with studies in fetal thymus organ cultures in which a complex mixture of class-I peptides has been shown to increase positive selection [68], [73]. Hence, it seems reasonable that fewer thymocytes will be positively selected in strains, such as DA.1HR10 and DA.1UR10, where the class I peptide repertoire is limited by the restrictive TAP-B transporter. In contrast to negative selection, positive selection is largely dependent on cortical thymic epithelial cells (cTECs). Interestingly, in the mouse it has been shown that thymic DCs express 10× higher levels of MHC class I than cTECs [74]. This latter subset is still poorly characterized in the rat and we have therefore not been able to determine the expression of class I on these cells. However, it seems likely that different allelic variants of *Tap2* would also affect the expression of class I on epithelial cells. The increased number of DP thymocytes should therefore be further investigated considering the lower expression of MHC class I described on cTECs in the mouse and the fact that the processing of antigens in these cells is different due to a thymoproteasome specific subunit encoded by *PSMB11* [75], [76].

Cim did not affect TCR selection at the early DN stage. All strains, regardless of *Tap2* genotype, had similar number of DN cells and normal transition from early thymic precursors to more mature TCRβ+ cells (DN4). By contrast, the frequency of early thymic progenitor cells was reduced in DA.1FR9, which is congenic for the *Tcs2* interval as well as the entire MHC-III region and parts of the non-classical MHC class Ib (RT1-CEM) region. A potential candidate in the MHC-III region, which could influence the regulation of DN cells in this strain, is *Notch4*. *Notch4* is expressed in the thymus [77] and has been shown to influence lymphoid progenitor fate [78] while another Notch family member, *Notch1*, has been shown to determine early commitment to T cell lineage [79]. We have recently obtained a new RCS, DA.1FR61 (Fig. 2), which excludes *Notch4* and future studies on this strain will help delineate whether *Notch-4* is involved in the regulation of DN cells.

We did not find evidence for a correlation between class I expression levels and T cell selection. All *Tcs1*-congenic strains showed low levels of extracellular class I expression (compared to DA) but varied widely in their CD8 cell numbers. Damoiseaux et al. suggested that the expression of two RT1-A genes in the BN strain, compared to one in LEW, was responsible for the increased negative selection of CD8 cells in the LEW.1N strain [36]. This reduction of CD8 cells is probably associated with the RT1-A locus (RT1-A^n^) in LEW.1N and thus the same phenotype as we mapped in DA.1IR85. We determined the expression of the two *RT1-A* genes (*A1^n^* and *A2^n^*) in DA.1IR85 and although both genes were highly expressed, the level of class I protein at the cell surface was low compared to DA. However, the low surface level of class I in DA.1IR85 is probably dependent on the presence of TAP-A in this strain, while RT1-A^n^ in LEW.1N is associated with TAP-B. Thus, it is unlikely that the reduction of CD8 cells in DA.1IR85 and LEW.1N [36] is associated with the levels of class I expression on the cell surface. However, that CD8 selection would be influenced by the number of protein-coding class I genes, as proposed by Damoiseaux et al. [36], remains an attractive hypothesis. It is further interesting to note that also DA.1HR83 expresses two RT1-A genes and has low numbers of CD8 cells, while DA and DA.1UR83, which have higher numbers of CD8 cells, only express a single RT1-A gene each. However, whether it is the number of expressed class I genes or the increased diversity of class I peptides that promote clonal deletion of CD8 cells remains to be tested in more strains.

We further noted that QTLs for the expression of class I (Q2 on RNO2, Q6 on RNO10) and class II (Q1 on RNO2, Q7 on RNO17) did not co-localize with QTLs for the CD4∶CD8 T cell ratio in the HS rats. Thus, *Tap2* remains, at least to our knowledge, the only naturally selected non-classical MHC gene that has been found to be associated with both variations in MHC surface expression and CD4∶CD8 lineage commitment. In addition to the MHC, a second major QTL controlling CD4∶CD8 T cell variation was identified within a narrow region on chromosome 9. A gene at this locus, *Satb1*, is predominantly expressed in the thymus [80]. Reduced expression of Satb1 at the protein level has been shown to be associated with a reduction in the frequency of CD8SP cells in the thymus as well as in the periphery [81]. Variation in CD8 T cells in the HS rats, however, did not correlate with the allelic distribution of *Satb1*, nor did the gene expression of *Satb1* in the thymus of 136 HS rats (data not shown).

A strong correlation similar to that we showed for *Tap2* and CD8 selection could not be found for any gene in *Tcs2* and CD4 selection. Apart from the extensive diversity we observed in *RT1-Db1*, *Ba* and *Bb*, several non-synonymous SNPs were in addition identified in *RT1-DOb*. This gene encodes one subunit of the non-classical class II molecule DO. The human homologue, HLA-DO, which has been shown to be expressed in the thymus [82], competes with the peptide editor HLA-DM for the binding of MHC class II molecules [83], [84]. Hence, polymorphisms that alter the affinity of DO for MHC class II may also affect the peptide repertoire of the MHC class II molecule and, thus, the selection of T cells. We found in addition evidence on transcript and protein level for a second RT1-Db locus (*Db2*), which is probably the rat ortholog of murine *Eb2* [85]. Similar to the mouse gene, *Db2* appears to be highly conserved among inbred rat strains. The genetic interaction between allotypes of TAP and RT1-A for the selection of CD8 T cells raises the question whether a similar interaction exists between the two highly polymorphic class II molecules (RT1-B and RT1-D) and alleles of *RT1-DOb* for the selection of CD4 T cells. Such interaction could be one explanation for the strong LD between these genes.

It has been a long-standing question why the rat has two TAP variants [24], in particular since the restrictive TAP-B transporter does not seem to offer advantages over the promiscuous TAP-A transporter. The redundancy of the TAP-B transporter has also been shown experimentally in the PVG.R23 recombinant strain in which RT1-A^u^, which is typically found associated with TAP-B, is expressed together with TAP-A [61]. In this strain, the association with TAP-A did not influence the expression of class I, which is consistent with our results in DA.1UR83. It was therefore somewhat surprising that certain other RT1-A allotypes appear to be dependent on the association with TAP-B for optimal expression on the cell surface. We termed this phenomenon *inverse* cim and showed that the extracellular expression of RT1-A^h^ is reduced when the molecule is associated with TAP-A compared to TAP-B. The same seems to be true for the RT1-A^n^ molecule(s) in DA.1IR85, although the linkage to TAP-A in the parental strain made it difficult to further address this question. One of the antibodies, F16-4-4, used to determine the inverse cim for the RT1-A molecules in DA.1HR83 has been shown to bind the A1^h^ but not the A2^h^ isoform (Anne France Le Rolle, personal communication). Thus, the inverse cim reduces the expression of A1^h^ while it remains to be investigated whether it also reduces the expression of A2^h^.

The expression of *RT1-A* at the gene level did not correlate well with the protein expression on the cell surface and did not explain the low levels of extracellular class I in DA.1HR83 and DA.1IR85. In DA.1HR83, the expression of *A2* was ∼15-fold higher compared to *A1*, which is inconsistent with the idea that if any allele is expressed at the *A1* locus, this allele will function as the principle class Ia molecule [18]. However, since both isoforms were detected at the protein level, it remains possible that A1 indeed is the dominant class Ia molecule in DA.1HR83, despite being expressed at a lower level. By contrast, *RT1-A1^n^* showed a 5-fold increased expression over *RT1-A2^n^* in DA.1IR85, and both isoforms were readily detected at the protein level. There was no evidence at the protein level for an A3 isoform, which may suggest that DA.1IR85 has not acquired all functional class Ia genes from BN. The class Ia molecules in DA.1IR85 further differed from DA.1HR83 by a high intracellular expression in lymphocytes, while a similar difference was not observed for myeloid cells. It should be noted that the OX18 antibody used for the intracellular staining of MHC class I also recognizes MHC class I in the absence of β2-microglobulin. Hence, the high intracellular levels of MHC class I in DA.1IR85 lymphocytes do not necessarily need to reflect an increase in mature peptide-loaded molecules. An interesting possibility is that the differences in intracellular class I levels between leukocyte lineages is regulated at the transcript level, which could suggest that the expression of *A1* and *A2* in DA.1IR85 is lineage specific. This has indeed been shown previously in rhesus macaques where specific class Ia transcripts have been associated with either myeloid or lymphoid lineages [86].

Co-evolution of genes in the class I and Tap loci is certainly not an isolated phenomenon in the rat, although the class I genes are separated from the TAP genes by the class II region in the majority of mammals. The class I molecules in both humans and mice have evolved to accommodate peptides that are supported by their associated TAP transporters, while the *Tap1* and *Tap2* genes themselves have remained functionally monomorphic. By contrast, functional alleles of *Tap2* that have co-evolved specifically with different alleles of class I have been described in the chicken [87]. The Tap genes in the chicken are directly flanked by class I genes [88], which probably have preserved the linkage. In the rat, by contrast, the *Tap2* and class I loci are separated by a ∼250 kb interval, which is relatively susceptible to recombinations (Fig. 3). On the contrary, the recombination activity between *Tap2* and the class II genes is extremely low as shown in this study. This may suggest that alleles of *Tap2* have been conserved in the rat in cis-configuration with alleles in the RT1-B and RT1-D loci. This linkage may not have been maintained in species that lack functional polymorphisms in the Tap genes, such as in the mouse [89]–[91] and in humans [92]. Highly conserved MHC-II haplotypes are also evident in many inbred and partially outbred rat strains [13], with the only exception to our knowledge being the WRC strain that has a recombination in the RT1-B locus [93]. In addition, several related inbred strains, which have been derived from outbred stocks, have recombinations between *Tap2* and *RT1-A*, e.g. LN (*Tap2B*) vs. LL (*Tap2A*), and FHL (*Tap2B*) vs. FHH (*Tap2A*) [13]. With an increasing number of genome sequences available, and the possibility to obtain sequence information from wild rats, it might be possible to investigate whether the strong linkage between *Tap2* and the class II genes is associated with certain haplotypes and bears an evolutionary advantage.

In summary, we mapped two QTLs associated with variations in CD4∶CD8 lineage commitment and MHC expression to the MHC-I and MHC-II region in the rat. A recombination between these two regions modifies class I expression by breaking the linkage between co-evolved *RT1-A* and *Tap2* alleles, which previously has been described as class-I modification (cim). We demonstrate a novel type of cim and also show that certain combinations of *RT1-A* and *Tap2* alleles are not affected by cim. Furthermore, we show that cim had a pronounced effect on thymic selection. Cim did not influence DN stages, but decreased the number of DP thymocytes. Most importantly, cim rescued CD8 T cells from negative selection and thereby increased the number of CD8 T cell in the periphery.

## Materials and Methods

### Ethics statement

All experiments involving animals were approved by the local ethics committees at Karolinska Institutet, Stockholm, or by the Spanish legislation on “Protection of Animals Used for Experimental and Other Scientific Purposes” and the European Communities Council Directive (86/609/EEC) on this subject.

### Animals

#### NIH-HS

The origin of the strains and the outbreeding regime that was used to create and maintain the NIH-HS have been described elsewhere [42]. Briefly, the colony was founded from 8 inbred progenitor strains: BN/SsN (RT1^n^), MR/N (RT1^d^), BUF/N (RT1^b^), M520/N (RT1^b^), WN/N (RT1^l^), ACI/N (RT1^a^), WKY/N (RT1^l^), and F344/N (RT1^lv1^) [37]. Animals were housed in open polycarbonate (Makrolon) cages at a temperature of 22°C±2°C and with 12 h light-dark cycle and fed standard rodent chow and tap water ad libitum.

#### Congenic strains

Inbred DA/Ztm rats were obtained from the Zentralinstitut für Versuchstierzucht (Hannover, Germany) and DA/OlaHsd from Harlan Europe (Horst, the Netherlands). Rats were maintained in a barrier facility by sister-brother mating and were specific pathogen free according to the current FELASA guidelines. Animals were kept in a climate-controlled environment with 14 h light/10 h dark cycles, in individually ventilated microisolator-cages (Allentown Inc. NJ, USA) containing wood shavings and fed standard rodent chow and microfiltered water ad libitum. Congenic strains were originally established on DA/Ztm background (N>20) and thereafter further backcrossed (N>5) to DA/OlaHsd. The RCS were produced by crossing F1 hybrid rats. The derivation of DA.1FR9, DA.1FR10, DA.1FR8 and DA.1FR5 from congenic DA.1F (DA.LEW-RT1^f^) has been described previously [94]. RCS with MHC haplotype RT1^u^ were derived from DA.1U, which was generated by introgression of the corresponding E3/ZtmRhd fragment on chromosome 20. RCS with MHC haplotypes RT1^i^ and RT1^h^ were derived from DA.1I and DA.1H, respectively (both established at the Zentralinstitut fur Versuchstierzucht). The RT1^i^ haplotype originates from the now extinct BI (formerly called B3) strain [95] and the RT1^h^ haplotype from the KHW strain. DA.1I contains a derivative MHC haplotype (RT1-A^i^B^a^D^a^); however, several STRs and SNPs in the MHC-II region and, to a lesser extent, in the MHC-III and Ib regions are polymorphic between DA.1I and DA. RT1^h^ is a standard haplotype [56] but most genetic variants in the MHC-II region are shared with RT1^n^ (this study). Several genes in the MHC-Ia region (including *RT1-A1* and *RT1-A2* [sequences from GenBank]) and the MHC-III region, however, are unique for RT1^h^ (Tuncel and Yau, unpublished data). Additional information about strains can be found on our website http://www.inflam.mbb.ki.se/rat/MHC. The QTLs described have identification numbers 7175096 (*Tcs1*) and 7175099 (*Tcs2*) in the Rat Genome Database (RGD) (http://rgd.mcw.edu/rgdweb/search/qtls.html).

### Genotyping

#### NIH-HS

Information on polymorphic SNPs was provided through the STAR consortium that identified SNPs and haplotypes in the rat to assist complex trait analysis as part of the EURATRANS consortium. DNA was extracted from liver biopsies using a standard proteinase K protocol. Genotyping was performed using a custom-designed high density Affymetrix SNP genotyping array (RATDIV), which is based on sequence information from 13 inbred rat strains. The array interrogates 803,485 SNPs of which 265,551 polymorphic high quality SNPs were chosen for the reconstruction of the HS chromosomes as a mosaic of the founder haplotypes as described below.

#### Congenic strains

Genomic DNA was extracted from biopsies as has been described previously [94]. PCR primers for short tandem repeats (STRs) and SNPs were retrieved from the rat genome sequence 3.4/rn4 2004 assembly and designed using PrimerSelect 8.1.3 (Lasergene, DNAstar Inc., WI, USA). Forward primers were fluorescently labelled with 6-FAM, HEX, VIC, PET or NED (MWG Biotech, Riskov, Denmark). STRs were amplified with PCR according to standard protocols, diluted in HPLC water, combined with the size standard Liz-600 in 10 µl HiDi formamide (both from Applied Biosystems, CA, USA) and analyzed on a 48-capillary 3730 DNA analyzer (Applied Biosystems). SNPs were genotyped by Sanger sequencing as described below.

### Exon sequencing

RNA was extracted from spleen using the RNeasy Mini kit (Qiagen, Ballerup, Denmark) and treated with DNase I (Roche, Mannhein, Germany). Complementary DNA (cDNA) was synthesized with iScript (Bio-Rad, CA, USA). Genomic DNA (gDNA) was isolated from spleen by proteinase K digestion (AquaPure Genomic DNA kit, Bio-Rad). Primer sequences were obtained from the RefSeq 3.4 genome assembly. All sequences were obtained through conventional capillary sequencing except *Btnl2*, which was sequenced on the SOLiD platform (Applied Biosystems). For capillary sequencing, products were amplified in a 25 µl PCR reaction containing 0.5 µg DNA, 2 µl 2.5 mM dNTP (New England Biolabs), 1 µl 50 mM MgCl2, 0.1 µl Platinum Taq (Invitrogen, CA, USA) and 0.5 µl of each 10 µM primer. The products were purified using the Millipore's Montage Cleanup Kit and diluted in HPLC water or were isolated by agarose digestion after gel electrophoresis. Sequencing was performed with BigDye Terminator v3.1 according to instructions (Applied Biosystems) using 0.4 µM of single primers. Products were purified by ethanol precipitation, resuspended in 10 µl HiDi formamide and analyzed on a 48-capillary 3730 DNA analyzer. Nucleotide sequences have been submitted to GenBank (http://www.ncbi.nlm.nih.gov/genbank) under accession numbers KC222882–KC222951.

### Flow cytometry

Analysis of PBMCs from NIH-HS rats has been described recently [41]. Briefly, phenotyping were performed in 8 cohorts of 230–270 individuals over 3 years. Rats had been subjected to behavioral tests (week 8–10) prior to blood sampling (week 13), but besides from a wound-healing test (puncture of the external ear) in week 7 and a glucose tolerance test (week 11), no invasive procedures had been performed. Twenty µl blood was stained for 20 min in duplicates with saturating concentrations of fluorescently-labeled monoclonal antibodies (MAbs, see below). After erythrolysis, cells were fixed for 20 min at RT in a 2% phosphate-buffered formaldehyde solution and then washed twice in PBS before acquisition.

#### Congenic strains

For analysis of MHC expression on DCs, tissues were cut into ∼1 mm^3^ cubes and incubated with 2 ml digestion buffer containing 2.5 mg/ml collagenase IV (Sigma-Aldrich, MO, USA), 0.2 mg/ml bovine pancreas DNase I (Roche Applied Science), 2% fetal calf serum (FCS, Gibco Laboratories, MA, USA) in Hank's Balanced Salt Solution (HBSS) (Sigma-Aldrich) for 20 min at 37°C. Undigested material was disrupted by pipetting, 2 ml fresh digestion buffer was added and the incubation was continued for 10–15 min. The digestion was stopped by adding EDTA (Merck) to a finale concentration of 20 mM. After incubation for 5 min in EDTA on an orbital shaker (100 rpm) at RT, the suspensions were filtered through 40 µm cell strainers (BD Falcon), and washed twice in ice-cold FACS buffer (calcium- and magnesium-free PBS-D supplemented with 1% FCS, 10 mM EDTA and 0.02% NaN_3_). For thymocyte and lymphocyte analyses, single-cell suspensions were prepared without collagenase/DNase I treatment, filtered through 40 µm cell strainers, washed twice in cold EDTA-FACS buffer and resuspended in the same buffer and counted on a Sysmex KX-21N. 10^6^ cells/well were added in duplicates to 96-well v-bottom polypropylene plates (BD Falcon) and stained with saturating concentrations of MAbs (30 µl finale staining volume). The following Alexa Fluor 488, FITC, PE, Pe-Cy5, PerCP-Cy5.5, APC, APC-Cy7, Pe-Cy7, Alexa Fluor 648 and biotin conjugated antibodies were used: CD4 (OX35), CD8a (OX8), CD8b (341), CD45RA (OX22), anti-granulcoytes (His48), RT1-B (OX6), RT1-D (OX17), MHC class I (OX18) were purchased from BD Pharmingen (CA, USA); CD45 (OX1), CD45RA (OX33), CD90 (OX7), CD4 (W3/25), CD11b/c (OX42), CD103 (OX62) and αβ-TCR (R73) were purchased from BioLegend (CA, USA); CD68 (ED1) and RT1-A (F16-4-4, conjugated in-house with Alexa Fluor 647 using the APEX labeling kit, Invitrogen) were obtained from AbD Serotec (Düsseldorf, Germany). CD2 (OX34) and CD5 (OX19) were produced in-house. To stain for β2-microglobulin (using clone TLD-3H12B, BD), as an alternative to OX18, was evaluated but did not result in reproducible data. R73 (αβ-TCR) was used to gate TCRβ+ cells in the DN population (Fig. 8). LIVE/Dead Violet (Invitrogen) was included in all stains to exclude necrotic cells. For the intracellular staining of class I, unlabeled OX18 or F16-4-4 was used to block extracellular epitopes and the cells were thereafter incubated in BD Cytofix/Cytoperm for 20 min at RT. Cells were then washed twice in BD PermWash and stained intracellularly with labelled OX18 and F16-4-4. A SORP BD LSRII Analytic Flow Cytometer or a FACSCalibur (BD) were used for acquisition and the data was analyzed with FlowJo (Tree Star Inc., OR, USA). Significant differences between groups were analyzed using a non-parametric test (Mann-Whitney U).

### Quantitative RT-PCR

RNA was extracted from 2×10^6^ non-stimulated spleen cells using the RNeasy Mini kit (Qiagen) and treated with DNase I (Roche). RNA concentration was determined spectrophotometrically on a NanoDrop ND-1000 (NanoDrop Technologies, DE, USA), diluted to 60 ng/µl and reverse transcribed to cDNA using High Capacity cDNA Reverse Transcription (Applied Biosystems). Quantitative real-time PCR (qPCR) was performed on an ABI 7900HT (Applied Biosystems) using SYBR Green (Applied Biosystems) and a two-step PCR protocol (95°C for 10 min followed by 40 cycles of 95°C for 10 sec and 60°C for 30 sec). Allele-specific primers (Table S3) for *RT1-A^a^*, *RT1-A^u^*, *RT1-A1^n^*, *RT1-A2^n^*, *RT1-A1^h^*, *RT1-A2^h^* and *clone 3.6* were designed using NCBI/Primer-BLAST with sequences retrieved from public databases. The performances of all primers were evaluated prior to use to ensure that all PCR reactions were performed at comparable efficiencies. In the final experiments, each target was amplified using 2–4 different primer pairs and the cycle threshold (Ct) values were then averaged at the gene level. Average Ct-values were compared to beta-2-microglobuline or normalized to the geometric mean of the reference genes *Arbp*, *Hprt-1* and *Mdh-1* (Table S3), whereafter fold-change variations were determined using the relative quantification method (ΔΔCt).

### Microarray

#### Array hybridization

Thymi and inguinal lymph nodes (iLN) were removed from DA.1IR83 rats and non-congenic littermates (n = 6) immediately post mortem. To minimize the risk of blood contamination in the thymi, the aorta was first incised below the chest to allow blood to drain into the abdominal cavity. Whole organs were excised, instantly frozen in liquid nitrogen and stored at −70°C until prepared. RNA extraction and array hybridization were performed as previously described [96]. Briefly, total RNA was extracted using TRIzol reagent, and further purified and DNase I treated using an RNeasy Mini kit (Qiagen) and RNase-Free DNase Set (Qiagen), according to the manufacturer protocols. RNA quality was assessed using the Agilent 2100 Bioanalyzer (Agilent Technologies, CA, USA). A total of 1 µg RNA was used for array hybridization against Affymetrix GeneChip Rat Exon 1.0 ST Arrays in accordance with the recommendations of the manufacturer (Affymetrix).

#### Data analysis

CEL intensity files were produced using GeneChip Operating Software version 1.4 (Affymetrix) and quality tested using the Affymetrix Expression Console. Detection of differential expression was performed at the gene level using the Partek Genomics Suite 6.4 (Partek Incorporated, MO, USA) and summarized at the gene level using a One-Step Tukey's Biweight Algorithm.

### Mass spectrometry

For the detection of class I derived peptides, single cell suspensions were prepared from spleens in PBS after lysis of erythrocytes. Cells were washed and taken up in 5% FCS supplemented DMEM-medium. 30 million cells were incubated for 7 hours at 37°C with 10 ng/ml IFN-γ, cells were washed in PBS and subsequently lysed in PBS containing 1.2% (w/v) CHAPS (GE Healthcare Life Sciences) and protease inhibitors (Complete, Roche Applied Science). RT1-D was immunoprecipitated from spleen cell-lysate using the monoclonal antibody OX-17. Proteins were reduced using 5 mM dithiothreitol (DTT) in 50 mM NH_4_CO_3_ for 10 min at 90°C and alkylated with 10 mM iodoacetamide (IAA) for 30 min at room temperature in the dark. Protein was precipitated overnight at −20°C in 95% acetone, precipitate was collected by centrifugation and washed with 80% acetone, 10% methanol, 0.2% acetic acid and incubated for 30 min at −20°C. Protein was pelleted by centrifugation and dissolved in 20 µl DMSO, diluted to 50 mM NH_4_CO_3_ and 30% DMSO with a final trypsin∶protein ratio of 1∶20 for in-solution digestion overnight at 37°C. Prior to MS analysis, the peptide mixture was dried, reconstituted in 5% formic acid, and cleaned using ZipTipC18 (Millipore) [97]. LC-MS/MS analyses were performed on an Easy-nLC system (Thermo Scientific, Bremen, Germany) directly on-line coupled to a hybrid QExcative Orbitrap mass spectrometer (Thermo Scientific). 1 µg of each sample was injected from a cooled auto sampler onto the LC column, peptide separation was performed on a 10 cm long fused silica tip column (SilicaTips, New Objective Inc., MA, USA) packed in-house with 3 µm C18-AQ ReproSil-Pur (Dr. Maisch GmbH, Ammerbuch, Germany). The chromatographic separation was performed using an acetonitrile (ACN)/water solvent system containing 0.2% formic acid with the following gradient set up: 5–35% ACN in 90 min, 35–95% ACN in 5 min and 95% ACN for 5 min all at a flow rate of 0.3 µl/min. The MS acquisition method consisted of one survey scan ranging from m/z 300 to m/z 1650 acquired in the FT-Orbitrap with a resolution of R = 70,000 at m/z 200, followed by ten consecutive data-dependent MS/MS scans from the top ten precursor ions with a charge state ≥2. The MS/MS scans were fragmented using HCD and acquired with a resolution of 17,500 at m/z 200. The instrument was calibrated externally according to the manufacturer's instructions and all samples were acquired using internal lock mass calibration on m/z 429.088735 and 445.120025. Mass lists were extracted from the raw data using the in-house written software Raw2MGF, and searched using the Mascot search engine (Matrix Science Ltd., London, UK) against a database consisting of the IPI rat database (v. 3.82), all accessions matching rat in the SwissProt database (downloaded 2011.04.05), as well as a protein FASTA file containing translated Sanger-sequenced regions of congenic MHC-II haplotypes (Fig. 4). The following parameters were used: tryptic digestion with maximum 2 miscleavages; carbamidomethylation of cysteines as a fixed modification; oxidation of methionine and glutamine to pyroglutamate conversions at peptide N-termini as variable modifications; precursor and fragment tolerance set to 10 ppm and 0.1 Da respectively.

### QTL mapping and statistical analysis

Genetic analysis was performed using HAPPY (http://www.well.ox.ac.uk/happy/) to calculate the probabilities of descent from the eight HS founders as described previously in [98]. To account for relatedness, the Efficient Mixed-Model Association eXpedited (EMMAX) method was used [41]. The negative logP threshold necessary to achieve a false discovery rate (FDR) of 10% across all traits was estimated by simulation to ∼4.2. To account for the complex family structure in the HS, we alternatively used Bagphenotype [99], where each QTL is scored by its Resample-based Model Inclusion Probability (RMIP). Confidence intervals were calculated by simulation of phenotypes, each arising from a single QTL, in addition to a correlated genetic random effect and uncorrelated errors. All the genome scans and annotated QTLs are publicly available from the website http://mus.well.ox.ac.uk/gscandb/rat.

## Supporting Information

Figure S1 The rat expresses two transcripts of *RT1-DOb*. (A) PCR amplification of cDNA from the 4 haplotypes tested with primers specific for *RT1-DOb* revealed two distinct products when separated by gel electrophoresis. (B) Analysis of the two products (shown in A) by Sanger sequencing revealed two transcripts of *RT1-DOb* (isf_1 and isf_2). The shorter transcript (Transcript Variant 2; GenBank accession number KC222928–KC222931) displayed a 77 bp deletion in exon 3 (red shaded area). (C) Amino acid sequence of *RT1-DOb* (haplotype RT1^av1^) with the 26-residue deletion in the β2 domain highlighted in red. (D) Structural model of rat *RT1-DOb* isoform 1 (haplotype RT1^n^, accession ID Q6MGA2). The red area illustrates the deletion in isoform 2 (Val 121-Thr146). (E) The shorter isoform of mouse *H2-Ob* (haplotype H2-g7, accession number Q3T9T7) lacks the cysteine, which corresponds to Cys143 in the long isoform of RT1-DOb, and can therefore not form a cysteine bridge with Cys173. See Figure S2 for the alignment of human, rat and mouse sequences.
(EPS)

Figure S2 MHC class II antigen DO beta chain alignment of human, mouse and rat. The 26 amino acid deletion identified on transcript level in rat *RT1-DOb* (see Figure S1) is also found in human *HLA-DOB* and mouse *H2-Ob*. All sequences aligned are based on evidence at the transcription level except for the 6 tryptic peptides (underscored), which were identified by MS in the proteome of B cells (see *Methods*). The tryptic peptides account in total for 32% coverage of the mature protein (isoform 1) but do not prove the existence of a shorter isoform. Residues marked with an asterisk (*) are shared in-between species. Transcript with accession number E9PND4 is a fragment, lacking exon 4–6.
(EPS)

Figure S3 Immunoprecipitation (IP) with anti-RT1-D alpha identifies RT1-Db2 specific peptides in cell lysate from DA.1HR61 and DA. RT1-Db2 was immunoprecipitated from DA (a) and DA.1HR61 (h) lymph node whole-cell lysate using the anti-rat RT1-D alpha specific antibody OX17. Identified peptides (pep1-6) that were identified in only one of the two lysates are marked as (a) or (h) whereas peptides that were identified in lysates from both strains are depicted as (a, h). Only peptides with Mascot scores >20 are shown. Identified peptides specific for RT1-Db1 are not shown in the alignment.
(EPS)

Figure S4 MHC class I molecules expressed in DA. Unique peptides identified in trypsin digested IFNγ-stimulated DA splenocytes matching MHC class I entries on UniProt. Peptide 9 is specific to RT1-A^a^ (HA12_RAT). Peptide 17 is specific to clone 3.6 (HA11_RAT) and the RT1-C^f^ entry O62936_RAT. Peptide 1–2, 4–8, and 10–12 match to HA12_RAT, but not to the non-classical HA11_RAT entry, while peptide 14–16 discriminate clone 3.6 from RT1-A^a^. Peptide 3 and 13 are shared between RT1-A^a^ and clone 3.6. Shaded amino acids indicate residues in the F pocket, which discriminate TAP-A from TAB-B-linked RT1-A molecules [24]. “Specific” indicates that the peptide matches only to the indicated entries on UniProt (other peptides also match to other class I entries, e.g. from other haplotypes).
(EPS)

Figure S5 MHC class I molecules expressed in DA.1UR83. Unique peptides identified in trypsin digested IFNγ-stimulated DA.1UR83 splenocytes matching MHC class I entries on UniProt. Peptide 2 is specific to the UniProt entries Q31256_RAT, Q95571_RAT, and Q95577_RAT, which all 3 refer to the gene RT1-A^u^. Q95571_RAT is identical to Q31256_RAT, but lacks the signal peptide. Q95577_RAT is identical to Q31256_RAT with the exception of a Val to Ala substitution at position 313. Peptides 1 and 3–5 are shared between these RT1-A^u^ entries as well as other rat MHC class I molecules. Shaded amino acids indicate residues in the F pocket, which discriminate TAP-A from TAB-B-linked RT1-A molecules [24]. “Specific” indicates that the peptide matches only to the indicated entries on UniProt (other peptides also match to other class I entries, e.g. from other haplotypes).
(EPS)

Figure S6 MHC class I molecules expressed in DA.1IR85. Unique peptides identified in trypsin digested IFNγ-stimulated DA.1IR85 splenocytes matching MHC class I entries on UniProt. Peptide 9 and peptide 8 are specific to the following UniProt entries Q6MGB9_RAT, E9PSS8_RAT, P79600_RAT, P79588_RAT, which are different length variants of RT1-A1^n^. Peptide 12 is specific to RT1-A2^n^ and matches to the UniProt entries P79602_RAT, E9PSX3_RAT, Q6MGB8_RAT, and F7ERG5_RAT, which are different length variants of RT1-A2^n^. Peptide 5, 10 and 11 further distinguish RT1-A1^n^ from RT1-A2^n^, but are also shared with other rat MHC class I entries. Peptide 1–3, 4 and 6–7 are shared between RT1-A1^n^ and RT1-A2^n^ as well as other rat MHC class I molecules. Shaded amino acids indicate residues in the F pocket, which discriminate TAP-A from TAB-B-linked RT1-A molecules [24]. “Specific” indicates that the peptide matches only to the indicated entries on UniProt (other peptides also match to other class I entries, e.g. from other haplotypes).
(EPS)

Figure S7 MHC class I molecules expressed in DA.1HR83. Unique peptides identified in trypsin digested IFNγ-stimulated DA.1HR83 splenocytes matching MHC class I entries on UniProt. Peptide 1 and peptide 7 are specific to RT1-A1^h^ (Q9QYQ3_RAT). Peptide 11 is specific to RT1-A2^h^ (Q9QYQ2_RAT) and peptide 13 only matches to the UniProt entries O62936_RAT and HA11_RAT. Peptide 4, 5, 8, 10 and peptide 12 further distinguish RT1-A1^h^ from RT1-A2^h^ or clone 3.6, while peptide 3 and 6 are shared between RT1-A1^h^, RT1-A2^h^ and the two class Ib molecules. Peptide 9 is shared between the two classical class I molecules, but not with clone 3.6. Shaded amino acids indicate residues in the F pocket, which discriminate TAP-A from TAB-B-linked RT1-A molecules [24]. “Specific” indicates that the peptide matches only to the indicated entries on UniProt (other peptides also match to other class I entries, e.g. from other haplotypes).
(EPS)

Figure S8 F16-4-4 recognizes a polymorphic determinant on MHC class Ia. The level of OX18 staining is ∼30-fold higher intracellularly than extracellularly, which probably reflects this antibody's affinity for MHC class Ib antigens. (A) DA splenocytes were stained with different concentrations of fluorescent-labelled anti-MHC class Ia and Ib (clone OX18) or anti-MHC class Ia (F16-4-4) antibodies. For detection of intracellular MHC class I, antibody epitopes on the cell surface were first blocked with unlabeled antibody. Cells were then fixed, permeabilized and stained intracellularly with fluorescent labelled antibodies using the same volume as used for extracellular staining. Histograms show samples with comparable mean fluorescent intensity when stained with indicated antibody dilutions (in brackets) extracellularly (red) and intracellularly (blue). (B) F16-4-4 binds a paraformaldehyde (PFA) sensitive epitope on MHC class Ia proteins. Non-permeabilized splenocytes from DA and DA.1IR85 were incubated with saturating concentrations of F16-4-4 (upper panel) or OX18 (lower panel) before (+ PFA) or after (− PFA) incubation of cells in a 4% PFA solution. Note the decreased F16-4-4 staining of PFA- treated cells from DA.1IR85, which suggests that F16-4-4 binds a polymorphic determinant (similar PFA sensitive epitopes were found in DA.1HR83 and DA.1UR83 (data not shown)). Gray histograms represent fluorescence minus one (FMO) controls.
(EPS)

Figure S9 Differentially expressed genes between DA and DA.1IR83 are confined to the *Tcs1* region. Microarray profile of gene expression in thymus (blue) and inguinal lymph nodes (purple) in DA.1IR83 and littermate DA rats (n = 6). Data show gene expression fold change levels at FDR <5% (above dashed line) and <10% (below dashed line). Asterisked genes are encoded within the congenic segment. Up - and downregulated genes in DA.1IR83 are shown on the right and left side of the vertical line, respectively. For the expression levels of classical MHC class I genes see Figure 6. Note that *Cd8a* and cathepsin W (*Ctsw*) are predominantly expressed in CD8 T cells and that the fold-change variation for these genes therefore is likely to reflect the variation in total number of CD8 T cells between the strains.
(EPS)

Figure S10 Progression of rat thymocytes from double negative to double positive stage. (A) Double negative (DN) cells in the rat are CD44+, similar to DN1, DN2 and DN3 in the mouse, but express no or very low levels of CD25 (IL-2 receptor; lower panel). (B) Triple negative (TN; CD4−, CD8−, TCRβ−) early thymic progenitor cells express CD45RC and CD5 (upper left panel) and low levels of CD2. CD2 expression increases when cells progress into double negative stage (CD2^hi^, CD45RC+). A small number of these cells stain positive with R73 (anti-αβTCR), indicating that they have started to rearrange and express TCRβ (upper right panel). More mature DN cells downregulate CD45RC to become CD2^hi^, CD45RC− (lower right quadrant). DN cells progress through an immature CD8α single positive stage (ISP). These cells have completed the rearrangement of the TCRβ locus and express low levels of TCRβ on the cell surface. Cells in the final DN stage, which correspond to DN4 in the mouse, express high levels of TCRβ on the cell surface. (C) Double negative cells progress into double positive stage. CD25 expression increases with the expression of TCRαβ on DP cells. Red arrows indicate the progress of maturation and are based on [63], [64].
(EPS)

Figure S11 Variation in CD4 T cell numbers decreases with thymic output. (A) CD4 T cells in the spleen were stained for CD90 and CD45RC (upper panel) or only for CD90 (lower panel) in two different experiments to determine the frequency of recent thymic emigrants (RTEs) in young (6 weeks of age) and old (13 weeks of age) DA and DA.1HR10 rats. The higher frequency of RTEs in young DA.1HR10 rats compared to young DA rats is not seen in the older rats and, hence, the variation in the total number of CD4 T cells (shown in B), seen in young rats, disappears when thymic output decreases in old rats.
(EPS)

Table S1 Polymorphic Short Tandem Repeat (STR) markers.
(DOC)

Table S2 Single Nucleotide Polymorphism (SNP) markers.
(DOC)

Table S3 Quantitative-RT-PCR primers.
(DOC)
