# Supplementary material for: Natural Polymorphisms in Tap2 Influence Negative Selection and CD4∶CD8 Lineage Commitment in the Rat
Source: PLoS Genet. 2014 Feb 20;10(2):e1004151. doi: 10.1371/journal.pgen.1004151 (PMC3930506; doi:10.1371/journal.pgen.1004151)
Supplement: Table S1 — Polymorphic Short Tandem Repeat (STR) markers. (DOC) [file pgen.1004151.s012.doc]

**Table S1 Polymorphic Short Tandem Repeat (STR) Markers**

| Marker ^a^ | Map No.^b^ | Position (Kb) ^c^ | Forward Oligo | Reverse Oligo |
| --- | --- | --- | --- | --- |
| D20Rat47 | 1 | 1616 | TGTCTCTTCAACCTCTCTGGC | AGGTTTGAGCCCCAGGATAC |
| D20RT1-M4 | 2 | 1633 | GCTGTGATTACCCTCACTGG | AGCTCCCGTACTAGAGTGTG |
| D20AA901295 | 3 | 2108 | GTTTAAAGCCTGAGTCTGCACG | AAGGGACAACGCTGGACAGTA |
| D20Wox5 | 4.0 | 2803+2780 | GAAAAATACTTCCACACACTAATG | AAAGTCAAGCCCTGGAGTG |
| D20Wox15 | 5 | 2791 | GAGAGGTGCCAGAAGATAGG | TGTCTCCCCAGTGTTGATTT |
| D20Wox3 | 5.1 | Not Mapped | AGGAAATGGGTTTCAGTTCC | CAGGATTCTGTGGCAATCTG |
| D20Arb2 | 6 | 2812 | CAGGAGGATTAAGAGAGGTGCC | CTGGCTTCAGAGATTCAACTGC |
| D20Mgh3 | 7 | 3430 | AACTTCCTGGTCCAGCCTG | CTCAGTGTGGAAGTGAGGAGC |
| D20Rat17 | 8 | 3394 | CGTTCGTGCGTCTGTCTTTA | AGCCCCAAATCCTCTGTTTT |
| D20TCL3547432 | 8.1 | 3547 | GATGGTGAGGTGACCCATGT | TTGTGTCCTATTTGCCTCCA |
| D20TCL3586149 | 8.2 | 3586 | CAGAGAGAGGCATGGAGAGG | TTCAAGGGTGAGGTTTCTGG |
| D20TCL3628068 | 9 | 3628 | TCCCTAGAGGCAGAAGGACA | GAGGGTAGTGATCCCACGAA |
| D20AY3635692 | 9.1 | 3636 | ACAGAGCCTGTGGGAAGATG | CTTCTGCCAGAGCAACAGTG |
| D20AY3649561 | 9.2 | 3650 | AGATGGGGATGGGGTACTTG | TGCAGGCATACTCCACCAC |
| D20AY3658172 | 9.3 | 3658 | CCTGTCTGTCCCCTTTTCTG | ATCTCTCTTGCTGCCTCACC |
| D20Uw1 | 11 | 3661 | GACAGAGAGGAAATGGGTTT | AATCTCCTCTCCGTCCTCAC |
| D20Wox2 | 12 | 3662 | GTTTCAGTTCTCAGGGTCCTA | CAGGATTCTGTGGCAATCTGG |
| D20TCL3735804 | 12.1 | 3736 | GTGTCAGTGAAGCCCTCTCC | AGACCTGCTGGAGAAAAGCA |
| D20Got4 | 13 | 3845 | CCAGGTCACCTCACACCATC | GAATTCCAGATTGTCCTTTGTCCT |
| D20AY3864471 | 13.1 | 3864 | AAGCCTTTGGTTTGGTTCC | GCCTCTGCGTGTCTTCCTAC |
| D20AY3905104 | 13.2 | 3905 | TCCTGCTCTCTGCCTTTCTC | CCAAGGGGAATGGTTAGTCC |
| D20Wox12 | 14 | 3957 | CTACATGAGCCCCTATTTGA | TCCACAGCAGGGAGTAATTG |
| D20TCL4046629 | 14.1 | 4047 | GCGAGACTTCCTTTCACCTG | GGAGAGAGCCAGCTTCTCAAT |
| D20TCL4102788 | 15 | 4103 | CAATTGATCGGTTAGCAGC | GAAAGAGAAAGACAGGGTAAGC |
| D20TCL4167082 | 16 | 4167 | GAACTGGGCCAAGAGTCAAC | CCTGGGCTGTAGAGTCTATGGT |
| D20TCL4209527 | 17 | 4210 | CAGTGTGGTACACATACGAACA | GCCAGCCTGGTCTACTTAAGA |
| D20AY4215171 | 17.1 | 4215 | GGGAGAACCTTCACACATGG | AGGGCTTCCTTTACCCTGAG |
| D20AY4223164 | 17.2 | 4223 | GGAATGGAACTGAGCCACAC | CCCCAAACACACACTGTCTTC |
| D20AY4229430 | 17.3 | 4229 | TCACTTGAGCCCAGGAGTTC | TATTGCTGGCATACGACTGG |
| D20AY4248866 | 17.4 | 4249 | GGCTGAAAGGAAACCTGTTG | CAGACCCCAGAACATGGAAG |
| D20AY4274359 | 17.5 | 4274 | TCCAGGATGAGACACTCACG | TGGGTGTGAGGTAAGGGAAG |
| D20TCL4300821 | 18 | 4301 | GGTCCGGAGTCCATTCTAGT | GTGATGGCCAAGAAACATAACT |
| D20Uia1 | 19 | 4571 | TTGAGACAGAATAGATAGGGCA | CCATTCATCAAATTCATGGTC |
| D20TCL4582614A | 20 | 4583 | TTCCAGTGGGCCAAGCAT | GCCCAGTTCTTCCACAGGTAAG |
| D20TCL4616388 | 21 | 4616 | ACTGGAGTAACTACCGTGG | AGCCTATAGGTAACCTAAGGAC |
| D20TCL4664906 | 21.00 | 4665 | TGCCTTTTCTGCACCTACTG | CCGCAACATAGACCAATATCC |
| D20TCl4670635 | 21.0 | 4671 | GAGAACTGGGGAAGGAGGAG | CTCGGGATGAGAGCTGTAGG |
| D20TCL4689077 | 21.05 | 4689 | TCTCTCTTCTGGCCTCCTTG | GGTCCCAGAAGGGATACACTG |
| D20TCL4716344 | 21.1 | 4716 | CTCTTCCAGCCTCTGTGGAC | TCCCAGCACCATTCTCTACC |
| D20TCL4738258 | 21.2 | 4738 | CCCCAAGCTCTCCTTAGTCC | GCAGCCTGCATTTCTTCTG |
| D20rat41 | 22 | 4741 | AGTCCTCTTCTGGCCTCCAT | TGGGACGATGTGTCATATCC |
| D20TCL481510 | 23 | 4815 | CAACAGTGTAGCGGAAGTTAG | TAAGCTGGTCATAGCATATCAA |
| D20TCL483895 | 24 | 4839 | CGTGGACGGAGGGTTTTAGAGT | CGACTGGGGATCCTATTGCTG |
| D20TCL485885 | 25 | 4859 | GGTGGATCTGTGTGAGTTCAA | CACCTGCTTCCCTTAACTATGA |
| D20TCL491775 | 26 | 4918 | TGACCTGATCTCGGCAAGA | GAAGACAGGGGGTGAGGATATT |
| D20TCL492275 | 27 | 4923 | GGATTGTCCAAAGCGTTACTAA | TTCCCTGTACCTCAACCCTTA |
| D20TCL495238 | 28 | 4952 | TGAGCAGAGAAAACATGAATAA | CCTCTCTCCTGGAATATATGTG |
| D20TCL497782 | 29 | 4978 | CACTCCAGGTTCACCCATGT | CTTGAAGTGAGAGCTGCAATGT |
| D20AA997966 | 31 | 5073 | AAGTTAACAGCCTTTATTAGAGAGTT | ACTTGTCCTCACAGCAGGGT |
| D20TCL5111233 | 32 | 5111 | GGGGTGTCATCTTCAAGTGTG | TAAGCTGCCACACACAAACC |
| D20TCL5113525 | 32.1 | 5113 | GGACCACAGTCTCATCAAAGC | TGCAGCAGTAGTCACACACG |
| D20TCL5131609 | 32.2 | 5131 | TGATACCCTCGTCTGGTGTG | CCTGTCCATCCCAATTAAGG |
| D20TCL5138331 | 32.3 | 5138 | GCCTCTAGACGCCATCTGTC | GCTTGCCTAAATCCCCAAC |
| D20TCL5139415 | 33 | 5139 | AACCTGGGTGTCCATCAGAG | GCAGGTGGATCTCTGAGTTTG |
| D20Rat45 | 34 | 5447 | AGGTCCTGTAGGACAGGGGT | ACCCCAGCAAGGAGAAAACT |
| D20TCL5916258 | 35 | 5916 | ACAAAGCAGGTCCCGTCA | GAGGCCATCAGATCCCATTATA |
| D20TCL5976714 | 36 | 5976 | CTGTCCTCAATCTCGGCTTGTG | GTGCCATCTGCCCTCGTG |
| D20Wox13 | 37 | 6696 | GTTTCTGTAATCCCATTTCTCTT | TTAGCAGTGCAAATTATCCAGA |

^a^ Novel markers are denoted TCL or AY; all other markers were retrieved from the Ensembl database. ^b^ Map numbers refer to positions shown in Figure 2 in the main paper. ^c^ Genomic coordinates were obtained from the UCSC Genome browser (3.4/rn4 2004 assembly).
