# Supplementary material for: Natural Polymorphisms in Tap2 Influence Negative Selection and CD4∶CD8 Lineage Commitment in the Rat
Source: PLoS Genet. 2014 Feb 20;10(2):e1004151. doi: 10.1371/journal.pgen.1004151 (PMC3930506; doi:10.1371/journal.pgen.1004151)
Supplement: Table S2 — Single Nucleotide Polymorphism (SNP) markers. (DOC) [file pgen.1004151.s013.doc]

Table S2. Single Nucleotide Polymorphism (SNP) Markers

|  |  |  | Allele | | | | |
| --- | --- | --- | --- | --- | --- | --- | --- |
| Gene/Region | Map No. ^a^ | Position (Mbp) ^b^ | RT1^a^ | RT1^i^ | RT1^f^ | RT1^u^ | RT1^h^ |
| *Btnl8* intron 1 | 4403 | 4,404,953 | A | nd ^c^ | nd | nd | G |
| *Btnl2* intron | 4618 | 4,618,296 | T | nd | nd | C | nd |
| *Btnl2/RT1-Da* (intergenic) | 4629 | 4,629,172 | C | nd | nd | C | T |
| *RT1-DMb* exon 2 | 4834 | 4,834,059 | C | nd | nd | nd | T |
| *RT1-DMb* 3'UTR | 4830 | 4,829,814 | G | nd | nd | nd | A |
| *Kifc1* exon 5 | 5161 | 5,160,774 | A | C | nd | nd | nd |
| *Syngap1* intron 1 | 5182 | 5,181,776 | G | A | nd | nd | nd |
| *Ggnbp1* exon 2 | 5267 | 5,266,580 | A | G | nd | nd | nd |

^a^ Map numbers refer to positions shown in Figure 2 in the main paper. ^b^ Genomic coordinates were obtained from the UCSC Genome browser (3.4/rn4 2004 assembly). ^c^ nd = not determined
