# Supplementary material for: Natural Polymorphisms in Tap2 Influence Negative Selection and CD4∶CD8 Lineage Commitment in the Rat
Source: PLoS Genet. 2014 Feb 20;10(2):e1004151. doi: 10.1371/journal.pgen.1004151 (PMC3930506; doi:10.1371/journal.pgen.1004151)
Supplement: Table S3 — Quantitative-RT-PCR primers. (DOC) [file pgen.1004151.s014.doc]

|  |  |  | **Primers** *^b^* | |
| --- | --- | --- | --- | --- |
| **Type** *a* | **Gene** | **Primer ID** | **Forward** | **Reverse** |
| HKG | *Beta-2-microglobulin* | 3_B2M | CGTGATCTTTCTGGTGCTTGTC | TTCTGAATGGCAAGCACGAC |
| HKG | *Acidic ribosomal phosphoprotein* | 182_Arbp | GCTTCATTGTGGGAGCAGACA | CATGGTGTTCTTGCCCATCAG |
| HKG | *Hypoxanthine-guanine phosphoribosyltransferase* | 178_Hprt-1 | GCGAAAGTGGAAAAGCCAAGT | GCCACATCAACAGGACTCTTGTAG |
| HKG | *Malate dehydrogenase* | 181_Mdh-1 | GATGGGTGTTCTGGACGGTG | GGAAGGGCACAGTCTTGCAG |
| HKG | *Hydroxymethylbilane synthase* | 180_Hmbs | TCTAGATGGCTCAGATAGCATGCA | TGGACCATCTTCTTGCTGAACA |
| Target | *RT1-Aa (allele specific)* | 247_RT1-Aa | GGAGTATTGGGAGCAGCAGA | CCTCAGGTCCACTCGGTAAA |
| Target | *RT1-Aa (allele specific)* | 257_RT1-Aa | TGGGAGCAGATTTACCGAGT | CCCTCGCTCTGGTTGTAGTA |
| Target | *RT1-A1i (allele specific)* | 274_RT1-A1i | TATTGGGAGGAGCAGACACG | GTCCTCAGGTTCACTCGGAA |
| Target | *RT1-A1i (allele specific)* | 275_RT1-A1i | AGAAACCGAGATACGAGCCG | GGTTCACTCGGAAAGTCTGC |
| Target | *RT1-A2i (allele specific)* | 31_RT1-A2i | CCAAGAGAAACGAGCAGGTTTAC | CCCCATGTCACAGCCATACA |
| Target | *RT1-A2i (allele specific)* | 270_RT1-A2i | TATTGGGAGAGGGAGACCCA | AGGTCCACTCGGTAAACCTG |
| Target | *RT1-A2i (allele specific)* | 272_RT1-A2i | TCCGCGGGTATTATCAGGAC | CCCTGAGTCTCTCTGCAACA |
| Target | *RT1-A1h (allele specific)* | 264_RT1-A1h | GGAGAATCCGAGGGTGGAG | CAGGTCCACTCGGAAAGTCT |
| Target | *RT1-A1h (allele specific)* | 265_RT1-A1h | TATTGGGAGCGGGAGACAC | CTCAGGTCCACTCGGAAAGT |
| Target | *RT1-A2h (allele specific)* | 266_RT1-A2h | AGATCACCCGGAACAAGTGG | CCTCCAGGTAGGCCTTTTCAT |
| Target | *RT1-A2h (allele specific)* | 267_RT1-A2h | ACGAAGACCTGAAGACGTGG | TCATAGTCTGCAGCACCTTCC |
| Target | *RT1-A2h (allele specific)* | 268_RT1-A2h | AGGTGCTGCAGACTATGAAAAG | TGACATCACCTTCAGGTCTGG |
| Target | *RT1-Au (allele specific)* | 269_RT1-Au | ACTTTGCTGCACAGATCACC | AATAATCTGCATCACCATCCCG |
| Target | *RT1-Au (allele specific)* | 244_RT1-Au | GATTACATCGCCCTGAACGAA | TGTAATAATCTGCATCACCATCCC |
| Target | *RT1-Au (allele specific)* | 245_RT1-Au | CGGATATAGGCAGGACGCC | TTGTAATAATCTGCATCACCATCCC |
| Target | *RT1-Au (allele specific)* | 246_RT1-Au | TCCTCCGCGGATATAGGCA | CTTGTAATAATCTGCATCACCATCC |
| Target | *Clone 3.6 (class Ib) (allele specific)* | 260_clone_3.6 | ACTTTGCAGCATGGATCACC | CCAGGTATCTGAGGAGCCAC |

**Table S3.** Quantitative-RT-PCR primers

*^a^* HKG, house keeping gene; *^b^* The following sequence accession numbers were used for primer-design: AJ249699.1 (RT1-A2h); AJ249698.1 (RT1-A1h); X82106.1 (RT1-Au); M31038.1 (RT1-Aa); X90376.1 (RT1-A2n); NM_001008827 (RT1-A1n)
